# Assessing intragenomic variation of the internal transcribed spacer two: Adapting the Illumina metagenomics protocol

**DOI:** 10.1371/journal.pone.0181491

**Published:** 2017-07-18

**Authors:** Lo’ai Alanagreh, Caitlin Pegg, Amritha Harikumar, Mark Buchheim

**Affiliations:** Department of Biological Science, The University of Tulsa, Tulsa, Oklahoma, United States of America; Chinese Academy of Medical Sciences and Peking Union Medical College, CHINA

## Abstract

Primary and secondary structural data from the internal transcribed spacer two (ITS2) have been used extensively for diversity studies of many different eukaryotic organisms, including the green algae. Ease of amplification is due, at least in part, to the fact that ITS2 is part of the tandemly-repeated rRNA array. The potential confounding influence of intragenomic variability has yet to be addressed except in a few organisms. Moreover, few of the assessments of intragenomic variation have taken advantage of the deep sequencing capacity of sequence-by-synthesis protocols. We present results from our adaptation of the 16S Metagenomics Sequencing Library Preparation/Illumina protocol for deep sequencing of the ITS2 genes in selected isolates of the green algal genus, *Haematococcus*. Deep sequencing yielded from just under 20,000 to more than 500,000 merged reads, outpacing results from recent pyrosequencing efforts. Furthermore, a conservative evaluation of these data revealed a range of three to six ITS2 sequence haplotypes (defined as unique sets of nucleotide polymorphisms) across the taxon sampling. The frequency of the dominant haplotype ranged from 0.35 to 0.98. In all but two cases, the haplotype with the greatest frequency corresponded to a sequence obtained by the Sanger method using PCR templates. Our data also show that results from the sequencing-by-synthesis approach are reproducible. In addition to advancing our understanding of ribosomal RNA variation, the results of this investigation will allow us to begin testing hypotheses regarding the maintenance of homogeneity across multi-copy genes.

## Introduction

The utilization of primary and secondary structures from the internal transcribed spacer two (ITS2) of the rRNA cistron has a long history in the field of molecular phylogenetics with over 15,000 citations for ITS2 phylogenetics returned in a recent query (Google Scholar) of a scientific literature that extends to the early 1990s. As part of the rRNA cistron, all copies of ITS2 are assumed to be subject to a homogenizing mechanism [1–5]. However, few investigators with a primary interest in phylogenetic analysis of ITS2 have explored this assumption. In the absence of data on intragenomic variability (IaGV), the validity of the ITS2 results is open to question [6–10]. Furthermore, studies have confirmed that IaGV is present or even common in at least some organisms [11–18].

A number of studies have used next-generation sequencing—largely pyrosequencing that targeted fungal diversity—to assess ITS2 variation [12, 19–28]. Of these investigations, we will focus on Song *et al*. [12] who used a pyrosequencing approach in an extensive survey of IaGV in the ITS2 of higher plants. Although considerable variation was detected (up to 253 nucleotide variants in one genome), the data indicated that intragenomic ITS2 variation in angiosperms manifests as a single dominant nucleotide variant and that variation does not generally confound studies of diversity [12, 29]. Moreover, other surveys of IaGV indicate that these data have tremendous potential to be exploited for diversity analysis [16, 20, 30–32].

**Fig 1:**
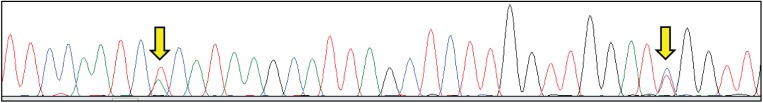
Electropherogram from Sanger sequencing of rRNA amplicons for a clonal isolate of *Haematococcus pluvialis*. Arrows indicate sites of putative intragenomic variation in a relatively short stretch of primary sequence.

Our interest in ITS2 diversity and evolution motivated us to address two questions that follow from our current understanding of IaGV. Are the angiosperm data regarding IaGV representative of other organisms in the Viridiplantae? In addition, we wondered if sequencing-by-synthesis methods could be exploited to obtain results at least comparable to pyrosequencing? We selected isolates of the green flagellate, *Haematococcus pluviali*s, to serve as our test organism for this investigation. Analysis of electropherograms from the sequencing of multiple, combined amplicons (Fig 1) led us to suspect that various isolates of *H*. *pluvialis* might have relatively high levels of IaGV compared to other taxa we have studied [33–36]. For this investigation, we adapted the 16S Metagenomics Sequencing Library protocol (Illumina) to investigate the nature of ITS2 variation in a small sampling of closely-related, green microalgae. Our results both confirm that IaGV exists in *Haematococcus* and validate the use of the Illumina system to assess IaGV by deep sequencing.

## Materials and methods

### Taxon sampling

Multiple distinct isolates of *Haematococcus pluvialis* from international culture collections (Sammlung von Algenkulturen, Göttingen) and from personal collections were included in the profiling of IaGV (Table 1). For this study, nine samples (isolates) comprising at least one representative from each of the six major lineages of *H*. *pluvialis* [35, 37] were included in the investigation.

**Table 1:** List of isolates studied and the quantitative tallies from results of deep sequencing for each isolate and replicate runs (R1, R2, or R3) for selected isolates.

Isolate of *H*. *pluvialis*	Filtered reads	Merged (single reads)
**SAG 34-1b R1**	2,247,678	591,186
**SAG 34-1b R2**	3,197,440	1,512,414
**SAG 34-1b R3**	3,230,078	1,644,994
**SAG 34-1c R1**	531,328	44,766
**SAG 34-1c R2**	3,012,714	1,176,852
**SAG 34-1c R3**	2,072,814	799,258
**SAG 34-1f R1**	993,916	111,240
**SAG 34-1f R2**	3,781,386	1,725,154
**SAG 34-1h R1**	660,812	35,178
**SAG 34-1h R2**	2,186,362	764,202
**SAG 34-1m R1**	601,442	31,258
**SAG 34-1m R2**	2,185,128	936,544
**SAG 49.94 R1**	987,942	82,684
**SAG 49.94 R2**	4,348,136	2,057,670
**SAG 44.96 R1**	720,308	19,396
**HP036 R1**	1,525,506	167,520
**HP111 R1**	3,513,038	1,296,446
**HP111 R2**	1,332,554	685,584

### DNA extraction

Genomic DNA was extracted using the E.Z.N.A plant DNA kit (Omega Bio-tek, Norcross, GA, USA) with some modifications to the manufacturer’s instructions. Approximately 50 ml of each sample culture was pelleted at 10,000 rpm in a 1.5 ml microcentrifuge tube. The pelleted cells were transferred to a screw cap microcentrifuge tube (2 ml) containing ~200 μl of autoclaved, 0.5 mm glass beads (Biospec Products, Bartlesville, OK, USA) and 600 μl lysis buffer from the kit. The cells were mechanically lysed using a Minibeadbeater (Biospec Products, Bartlesville, OK, USA) set at maximum speed for 10–30 seconds. The remainder of the extraction followed the kit protocol instructions. The quantity of DNA was determined using a Qubit 2.0 Fluorometer (Thermo Fisher Scientific, Waltham, MA, USA). A dilution of 5 ng/μl was prepared for each sample to be used in PCR reactions.

### Primer design and amplicon generation

We adapted the 16S metagenomics protocol by Illumina for our assessment of IaGV. Instead of targeting the V3 and V4 regions of the 16S rRNA from bacterial taxa, we designed specific primers (forward and reverse) to amplify the ITS2 regions of *Haematococcus*. The primers ITS2-F2 (5’- GCA TAT TGC GCT CAA GGC TTC GG -3’) and ITS2-R2 (5’- TCC TCC GCT TAT TGA TAT GCT TAA GTT CAG CG -3’) were tested and adapted for this study. After successfully testing functionality, both primers were tagged with adapters following the Illumina protocol. Specifically, a forward overhang sequence (5’TCG TCG GCA GCG TCA GAT GTG TAT AAG AGA CAG-[locus specific sequence]) and a reverse overhang: sequence (5’GTC TCG TGG GCT CGG AGA TGT GTA TAA GAG ACA G-[locus specific sequence]) were added to the primers during primer synthesis. The customized primers were used to amplify ITS2 regions from all *H*. *pluvialis* samples. All PCR was performed in a 25 μl reaction containing 12.5 μl 2x KAPA HiFi HotStart ReadyMix (Kapa Biosystems, Wilmington, MA, USA) and 12.5 ng genomic DNA containing 5 μM of the forward and reverse primers. Thermal cycling conditions were initiated by denaturation at 95°C for 3 minutes followed by 25 cycles at 95°C for 30 seconds, 55°C for 30 seconds and 72°C for 30 seconds. The program ended with a final extension at 72°C for 5 minutes. Following amplification, 1 μl from each PCR product was used to verify the size of the amplicons on an Agilent 2100 Bioanalyzer using a Bioanalyzer DNA 1000 chip (Agilent Technologies, Santa Clara, CA, USA).

### Illumina MiSeq (sequencing-by-synthesis)

The Illumina sequencing libraries were generated using a Nextera XT Index kit (Illumina, San Diego, CA, USA) following manufacturer’s instructions. Following the first stage PCR to amplify the ITS2 region (amplicon generation), the ITS2 amplicons were purified and indexed independently using the Nextera XT Index kit by running second stage PCR (PCR conditions were applied as provided by the protocol). After indexing, the samples (DNA libraries) were subjected to a final purification step and the quality and quantity of the libraries were assessed using the Qubit 2.0 Fluorometer and the Agilent 2100 Bioanalyzer (Agilent Technologies, Santa Clara, CA, USA). Finally, the libraries were pooled and sequenced with an Illumina MiSeq platform at the University of Tulsa using the 2x300 bp paired end protocol (MiSeq Reagent Kit v3-600 cycles; Illumina, San Diego, CA, USA).

### Sequencing data analysis

Raw sample reads were filtered using the FASTQ toolkit (Illumina BaseSpace Labs). The filtration parameters included a minimum read length of 250 bp and a minimum QC value of 30. In addition, the sequence passages corresponding to the adapters were trimmed from all reads. The filtered reads were processed using the CLC Genomics Workbench (Qiagen, Germantown, MD, USA), where pairs of overlapping reads were merged. A total of 10,000 reads were randomly selected from each set of filtered and merged reads following the mapping step. All merged and sampled reads were mapped to a specific ITS2 reference (*e*.*g*., the published ITS2 sequence for the SAG 34-1f isolate of *H*. *pluvialis* served as the map for all reads from the sequencing of amplicons generated from templates of the SAG 34-1f isolate). Results from five replicates of the random sampling plus mapping protocol were averaged for each template. Unique ITS2 variants (*i*.*e*., unique ITS2 sequences) were identified as discrete haplotypes. Thus, haplotypes were defined as unique sets of nucleotide polymorphisms (SNPs and DNPs) and indels rather than each unique nucleotide polymorphism or indel serving as a discrete variant or haplotype. We chose to define the sequence as the unit variant since it is the sequence that is the fundamental unit in phylogenetic analysis. Only nucleotide polymorphisms or haplotypes with frequency greater than or equal to 1% of the sampled sequences were treated as demonstrable variants. These averaged sets of randomly sampled reads were used to generate estimates of relative nucleotide polymorphism frequency and haplotype frequency.

### *P*-distance analyses

MEGA7 [38] was used to calculated values of mean *p*-distance for intra- and inter-lineage comparisons (all possible pairwise comparisons) and intra- and inter-isolate comparisons (within the *H*. *pluvialis* or “A” lineage [37]). Nucleotide alignments of ITS2 data for these analyses were guided by secondary structure as implemented in 4Sale v 1.7 [39, 40] (see also below).

### Reproducibility

Reproducibility was assessed by conducting replicate deep sequencing runs for several isolates. Replicates included reamplification of a single template and re-extraction of selected templates for replicate sequencing.

### Cloning

As further validation, several templates (SAG 34-1b, SAG 34-1h, SAG 34-1m, SAG 34-1f, SAG 44.96, SAG 49.94, and HP036) were selected to serve as the basis for cloning and sequencing of the ITS2 gene. Amplicons targeting the transcribed spacer were generated by PCR [35] and then purified using illustra™ GFX™ PCR DNA and Gel Band Purification Kits (GE Healthcare, Buckinghamshire, UK) for use in cloning reactions. Cloning was carried out using a TOPO TA Cloning Kit (Invitrogen, Carlsbad, CA, USA). Ligation conditions followed the manufacturer's protocol as optimized for high transformation with minimal ligation time (5 min). All reactions were conducted at room temperature. In order to increase reproducibility of the results, plasmid DNA was isolated from positive colonies using the ChargeSwitch-Pro Plasmid MiniPrep kit (Invitrogen, Carlsbad, CA, USA).

### DNA sequencing

Plasmid DNA from each clone was prepared for Sanger sequencing using standard vector-specific M13 forward and reverse primers. All cycle-sequencing reactions were carried out using an Eppendorf Gradient Thermal Cycler (Brinkman Instruments, Westbury, NY, USA). Sequencing reactions were completed using the reagents and protocols for BigDye v3.1 (ABI, Foster City, CA, USA). All sequencing was conducted using an ABI 3130xl Genetic Analyzer (ABI, Foster City, CA, USA).

### Sequence assembly and editing

Raw data from both strands of the sequenced clones were assembled and edited using Sequencher v 4.9 (Gene Codes Corporation, Ann Arbor, Michigan, USA). Exemplars from cloned sequence variants and from deep sequencing variants were aligned (sequence only) as described below. Comparison of sequences was conducted using Mesquite v. 3.2 [41].

### Phylogenetic reconstruction

Exemplars for all unique haplotypes from deep sequencing (see Supporting information S1 to S9 Figs) and from cloning were assembled for phylogenetic analysis. Published reference sequences from each of the isolates were included in the phylogenetic analyses. Published ITS2 sequence data from *Gungnir neglectum* and *Chlamydomonas applanata* [42] were used to root the trees. Secondary structure for each haplotype sequence was determined by homology-modeling using the tools in the ITS2 Database V [43–45], in which a published XFASTA file [35, 42], corresponding to each isolate group, served as the template. Final alignment for ITS2 sequence-structure analysis was completed using 4Sale v 1.7 [39, 40]. The set of aligned XFASTA files for the ITS2 data were subjected to sequence-structure (SS) analysis using the Neighbor-Joining algorithm (with GTR distances generated using the Q-ITS2 12x12 rate matrix) as implemented in ProfDistS [46–49]. Bootstrap values [50] from 500 replicates were generated to assess the relative strength of signal from the sequence-structure data. A sequence-only data set was obtained by export from 4Sale v. 1.7 [39, 40]. The sequence-only data were analyzed to identify the best fit model of nucleotide substitution using PAUP v4.0a152 [51]. Neighbor-Joining (NJ) and Maximum Likelihood (ML) trees were generated using the SYM+G (G = 1.95543) model parameters as implemented in PAUP v4.0a152 [51]. For the ML analysis, 10 random addition sequence replicates were conducted in a heuristic search of treespace using the TBR branch-swapping algorithm. A total of 1000 replicates were generated for the bootstrap analysis that accompanied the NJ analysis and a total of 100 replicates were generated for the bootstrap analysis that accompanied the ML analysis. For the ML bootstrap analysis, all starting trees were constructed by the NJ method and branch-swapping was conducted using the NNI branch-swapping algorithm.

## Results

### Deep sequencing

Raw reads from deep sequencing using the modified Illumina protocol for 16S metagenomics analysis varied from 1.2 million to more than 3 million. Filtered reads ranged from a little more than 500,000 to more than 2.2 million (Table 1). Merged reads ranged from just under 20,000 to nearly 600,000 (Table 1). The frequency of the numerically dominant haplotype ranged from approximately 0.35 to 0.98 (S1 to S9 Figs). Most variants were characterized by base substitutions, but some indels were also observed (S1–S5 Figs). The numerically dominant haplotype corresponded to the published ITS2 sequence in all relevant cases (S2–S8 Figs) except for the SAG 34-1b (S1 Fig) and HP111 (S9 Fig) where two ITS2 haplotypes were observed with nearly equivalent frequencies. For all but the SAG 34-1b isolate (S1 Fig), at least one variant site that was detected by deep sequencing (S1–S8 Figs) could also be identified as ambiguous in the raw electropherograms upon which the published Sanger sequences were based (S11–S18 Figs). Evidence for an indel was observed in a portion of the electropherograms used to assemble Sanger sequences for SAG 34-1m (S14B Fig).

### *P*-distances

Results from distance analysis (*p*-distances) showed that within lineage variation (Table 2) ranged from less than 0.008 (RUBICUNDUS lineage) to more than 0.04 (RUBENS lineage). Between lineage variation (Table 3) ranged from less than 0.045 (RUBICUNDUS vs Lineage C) to more than 0.15 (PLUVIALIS vs Lineage D). Within lineage variability (Table 2) was, on average, nearly one order of magnitude lower than between lineage variability (Table 3). Statistical analysis (i.e., a students T-test) confirmed that the mean values for within-lineage variability and between-lineage variability are significantly different (*P* = 1.32 x 10^−7^). Average within isolate variability (Table 4) was lower than between isolate variation (Table 5), but the difference between the two sets of data was not statistically significant (*P* = 0.48).

**Table 2:** *P*-distances for within-group (lineage) comparison of ITS2 haplotypes.

Lineage	Within Group Mean *P*-Distance
**PLUVIALIS**	0.011461282
**RUBICUNDIS**	0.00763203
**C Lineage**	0.022749559
**RUBENS**	0.040381791
**D Lineage**	0.007980508
**B Lineage**	0.009270965
**Mean**	0.016579356

**Table 3:** *P*-distances for between-group (lineage) comparison of ITS2 haplotypes.

Lineage 1	Lineage 2	Between Group Mean *P*-Distance
**PLUVIALIS**	**RUBICUNDIS**	0.101
**PLUVIALIS**	**C Lineage**	0.088
**RUBICUNDIS**	**C Lineage**	0.042
**PLUVIALIS**	**RUBENS**	0.043
**RUBICUNDIS**	**RUBENS**	0.091
**C Lineage**	**RUBENS**	0.076
**PLUVIALIS**	**D Lineage**	0.167
**RUBICUNDIS**	**D Lineage**	0.126
**C Lineage**	**D Lineage**	0.118
**RUBENS**	**D Lineage**	0.155
**PLUVIALIS**	**B Lineage**	0.116
**RUBICUNDIS**	**B Lineage**	0.104
**C Lineage**	**B Lineage**	0.093
**RUBENS**	**B Lineage**	0.097
**D Lineage**	**B Lineage**	0.153
	**Mean**	0.105

**Table 4:** *P*-distances for within-group (isolate) comparison of ITS2 haplotypes for isolates affiliated with the *H*. *pluvialis* (A) lineage.

Isolate	Within Group Mean *P*-Distance
**SAG 34-1b**	0.011758569
**HP111**	0.008810573
**SAG 49.94**	0.011804383
**Mean**	0.010791175

**Table 5:** *P*-distances for between-group (isolate) comparison of ITS2 haplotypes for isolates affiliated with the *H*. *pluvialis* (A) lineage.

Isolate 1	Isolate 2	Between Group Mean *P*-Distance
**SAG 34-1b**	**HP111**	0.012467350
**SAG 34-1b**	**SAG 49.94**	0.010637853
**HP111**	**SAG 49.94**	0.011981126
	**Mean**	0.011695443

### Deep sequencing vs. cloned sequences

**Fig 2:**
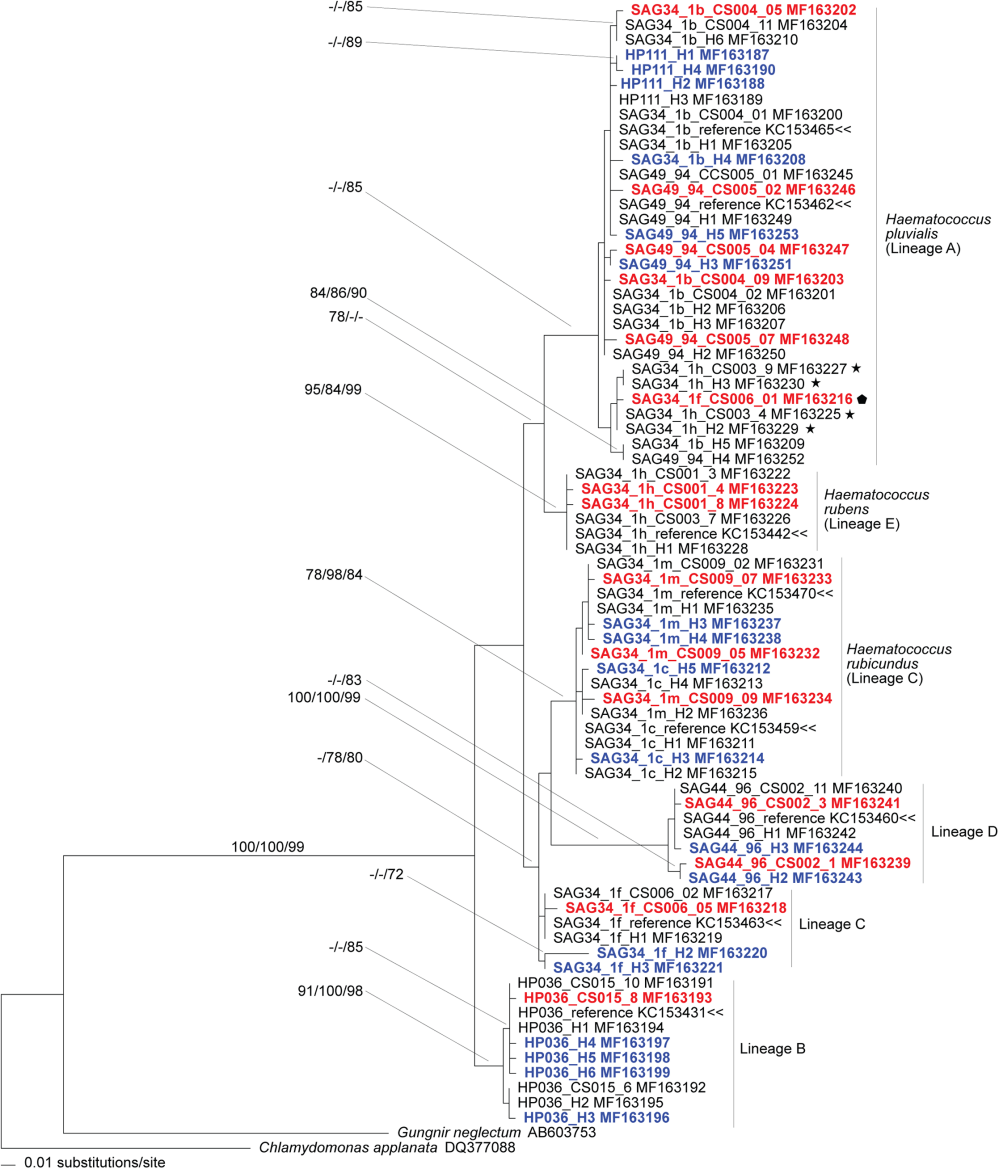
Results from phylogenetic analysis of data from intragenomic ITS2 variants (i.e., haplotypes) of *Haematococcus*. Reconstruction is from ML analysis of alignment guided by secondary structure. Branch lengths are drawn proportional to evolutionary change as recorded by the ML analysis. Bootstrap values from NJ, ML and SS are mapped (in that order) to corresponding branches. Lineage labels are from Allewaert *et al*. [37] and Buchheim *et al*. [35]. Haplotypes from deep sequencing are denoted with “H” followed by a number. Haplotypes from cloning are denoted with “CS” followed by a number. Published ITS2 sequences that were used as references are denoted by a double arrowhead (<<). The four, haplotypes of SAG 34-1h (*H*. *rubens* lineage) that are allied with the *H*. *pluvialis* (A) lineage are highlighted with stars. The cloned haplotype of SAG 34-1f (Lineage C) that is allied with the *H*. *pluvialis* (A) lineage is highlighted with a pentagon. Haplotypes with unique combinations of nucleotide variants (relative to the reference) are presented in red (clone and sequence) or blue (deep sequenced) boldface.

Sequencing of cloned amplicons yielded similar, but not wholly identical results from deep sequencing. In all but one case (SAG 34-1h; Table 6), deep sequencing yielded at least as many ITS2 haplotypes detected by cloning. However, a few additional cloned haplotypes from isolates other than SAG 34-1h were distinct from the haplotypes detected by deep sequencing (see Fig 2).

**Table 6:** Variants of ITS2 (cloned and deep sequenced) with number of haplotypes from cloning and deep sequencing and total number of nucleotide polymorphisms.

Lineage & Isolate	Number of Cloned Sequences	Total Number of Cloned ITS2 Haplotypes	Total Number of Deep Sequenced ITS2 Haplotypes	Total Number of Deep Sequenced Nucleotide Polymorphisms
**PLUVIALIS Lineage (*Haematococcus pluvialis* SAG 34-1b)**	9	5	6	9
**RUBICUNDUS Lineage (*Haematococcus rubicundus* SAG 34-1c)**	N/A	N/A	4	5
**Lineage C (*Haematococcus pluvialis* SAG 34-1f)**	11	3	3	10
**RUBENS Lineage (*Haematococcus rubens* SAG 34-1h)**	18	6	3	14
**RUBICUNDUS Lineage (*Haematococcus rubicundus* SAG 34-1m)**	10	4	4	6
**PLUVIALIS Lineage (*Haematococcus pluvialis* SAG 49.94)**	10	4	5	7
**Lineage D (*Haematococcus pluvialis* SAG 44.96)**	11	3	3	3
**Lineage B** **(*Haematococcus pluvialis* HP036)**	8	3	6	6
**PLUVIALIS Lineage** **(*Haematococcus pluvialis* HP111)**	N/A	N/A	4	3

### Reproducibility

Replicate deep sequencing runs resulted in identical patterns of variant analysis in terms of numbers of haplotypes detected (Tables 7 and 8; S1–S6 and S9 Figs). The dominant haplotype was identical for all replicate tests (S1–S6 Figs) except for the HP111 replicates (S9 Fig). In the latter, the two most common haplotypes demonstrated frequency values less than 50% (S9 Fig). Absolute values for haplotype frequency showed variability across replicates (Tables 7 and 8).

**Table 7:** Comparison of haplotype frequency calculations from replicate deep-sequencing for three isolates from the *H*. *pluvialis* (A) lineage.

	SAG 49.94		Mean	SD	SE	SAG 34-1b			Mean	SD	SE	HP111		Mean	SD	SE
	Haplotype Frequency					Haplotype Frequency						Haplotype Frequency				
**H1**	0.57#	0.60#	0.586	0.022	0.016	0.39#	0.35#	0.35*	0.363	0.024	0.0137	0.43#	0.39#	0.408	0.0304	0.0215
**H2**	0.28#	0.26#	0.271	0.015	0.011	0.34#	0.3#	0.32*	0.322	0.023	0.0130	0.42#	0.45#	0.436	0.0247	0.0175
**H3**	0.07#	0.07#	0.069	0.005	0.004	0.17#	0.28#	0.27*	0.238	0.060	0.0346	0.14#	0.14#	0.143	0.0021	0.0015
**H4**	0.05#	0.05#	0.048	0.002	0.002	0.05#	0.04#	0.04*	0.045	0.008	0.0046	0.01#	0.02#	0.015	0.0028	0.002
**H5**	0.03#	0.03#	0.027	0.000	0.000	0.03#	0.02#	0.01*	0.020	0.009	0.0049	x	x	x	x	x
**H6**	x	x	x	x	x	0.01#	0.01#	0.01*	0.012	0.002	0.0012	x	x	x	x	x

Replicates from the same template are indicated by #.

Replicates derived from a new template are indicated by *.

H = haplotype. SD = standard deviation. SE = standard error of the mean.

**Table 8:** Comparison of haplotype frequency calculations from replicate deep-sequencing for two isolates from the *H*. *rubicundus* lineage.

	SAG 34-1m		Mean	SD	SE	SAG 34-1c			Mean	SD	SE
	Haplotyype Frequency					Haplotype Frequency					
**H1**	0.67#	0.762#	0.716	0.065	0.046	0.67#	0.65#	0.63*	0.649	0.019	0.0112
**H2**	0.288#	0.245#	0.267	0.030	0.022	0.27#	0.28#	0.32*	0.290	0.029	0.0166
**H3**	0.028#	0.033#	0.031	0.004	0.003	0.03#	0.03#	0.02*	0.027	0.007	0.0043
**H4**	0.015#	0.018#	0.017	0.002	0.002	0.02#	0.02#	0.02*	0.020	0.004	0.0021
**H5**	x	X	x	x	x	0.01#	0.01#	0.02*	0.014	0.002	0.0009

Replicates from the same template are indicated by #.

Replicates derived from a new template are indicated by *.

H = haplotype. SD = standard deviation. SE = standard error of the mean.

### Phylogenetic reconstruction

Results from phylogenetic analysis of the ITS2 haplotypes (from cloning and deep sequencing) and published ITS2 sequences (Sanger sequencing) sorted all the sequence variants into lineage-specific clusters (Fig 2) with two notable exceptions concerning isolates SAG 34-1f (portion of lineage “C”) and SAG 34-1h (*H*. *rubens*). Branches supporting four of the five lineages of *Haematococcus* exhibited modest to strong bootstrap support (Fig 2) by at least one of the reconstruction methods (*H*. *pluvialis* [lineage “A”], lineage “B”, *H*. *rubicundus* [portion of lineage “C”], lineage “D” and *H*. *rubens* [lineage “E”]). That portion of lineage “C” that comprises sequence variants from the SAG 34-1f isolate was not strongly supported by any method (Fig 2). Furthermore, one of the cloned haplotypes of SAG 34-1f (CS006-01) was allied with the *H*. *pluvialis* (lineage “A”) clade (Fig 2). Although the *H*. *rubens* alliance (lineage “E”) enjoys strong bootstrap support, four haplotypes (3 deep sequencing and 1 clone) derived from the *H*. *rubens* isolate (SAG 34-1h) were allied with the *H*. *pluvialis* (lineage “A”) clade (Fig 2). These data also corroborate the observation that lineage “C” [35] is comprised of two distinct lineages, one of which is now regarded as *H*. *rubicundus* [37]. The bulk of SAG 34-1f haplotypes comprised the other “C” lineage (Fig 2). Phylogenetic relationships among the lineages of *Haematococcus* were not well-resolved by the data, but these results are not fundamentally different from previous studies based on dominant haplotypes [35, 37, 42]. Any differences in topology among the various results correspond to branches that lack robust support.

## Discussion

### Deep sequencing vs Sanger sequencing

Careful study of the electropherograms that were originally used to generate the relevant published ITS2 sequences [35] revealed evidence of IaGV in the form of both substitutions and indels. However, not all relevant Sanger sequences showed unambiguous evidence of base-call ambiguity when variant sites were detected by deep sequencing (cf. S10 and S11 Figs). Furthermore, one subordinate variant from Sanger sequencing did not correspond to the subordinate variant detected by deep-sequencing (S17 Fig). These observations indicate that Sanger sequencing may provide insight into IaGV, but should not be relied upon as evidence for all examples of IaGV.

### Reproducibility

The results of our experiments indicate that the Illumina method is reproducible for use in identifying haplotype variants of ITS2. Our data revealed variability for the absolute value of haplotype frequency (and nucleotide variant frequency), but no differences were observed for the relative frequency of haplotypes across replicates except for the HP111 isolate (see below). While the absolute frequency values show some variability, the magnitude of the variability is generally less than 5%. Furthermore, values for the standard error of the mean (Tables 7 and 8) indicate that the variability in frequency values would not impair our ability to discriminate between results from distinct templates. Future experiments will help us determine whether the variability in absolute haplotype frequency is a product of experimenter error (*e*.*g*., slight differences in pipetting efficiency from one experiment to another). We must also be able to separate experimenter error from the possibility of actual frequency variation. When comparing data from two different extracts of the same isolate, any differences in absolute frequency may be dependent on actual changes in the frequency of haplotype copies within the genome. Since these isolates are maintained in clonal culture, the continued pattern of cell division in the absence of a mechanism for homogenization (i.e., unequal crossing-over during meiosis) could lead to changes in absolute or relative haplotype frequency. Thus, our future experimental design must include multiple amplicons from the same template as well as amplicons from multiple extracts for the same isolate. Ideally, the former should not manifest any substantive differences in absolute variant frequency while variation in the latter could be a consequence of a real biological phenomenon. We conducted a simple analysis of the two samples (SAG 34-1b and SAG 34-1c) for which we have both types of replicate data (new amplicons from the same template and amplicons from a new extract). These analyses showed that the mean of the squared differences in haplotype frequency was greater for differences between haplotypes derived from two different extracts than for parallel differences between haplotype frequencies derived from the same DNA extract (data not shown). While it is tempting to conclude that the differences in IaGV frequency for different extracts may reflect actual biological diversity, the sample size was insufficient to do much more than note the trend.

Results from analysis of the HP111 isolate (S9 Fig) indicated that the dominant haplotype was different in the two replicates. The difference in frequency value between the two haplotypes was close to the average difference noted for other replicate frequency values (ca. 0.04). Thus, the truly distinctive feature about the HP111 isolate was that it appears to have co-dominant haplotypes. Examination of the electropherogram from Sanger sequencing of PCR products showed overlapping “C” and “T” peaks of nearly equal magnitude (S18 Fig). All other isolates examined in this investigation were characterized by a single dominant haplotype. Although a published Sanger sequence is not currently available for comparison with the results of the deep sequencing, an unpublished Sanger sequence was created to serve as a reference for HP111.

### Illumina sequencing vs pyrosequencing

Our results demonstrated that the Illumina protocol is capable of greater depth of sequencing than 454/pyrosequencing for this unusual application of deep sequencing. Our results yielded a low of 19,000 merged reads and a high of nearly 600,000 merged reads. In contrast, pyrosequencing yielded only a few thousand reads [12]. Comparing our IaGV/ITS2 results for *Haematococcus* with the results of the Song *et al*. [12] study of IaGV/ITS2 in flowering plants is problematic given the different taxa and methodologies that were used. At face value, the maximum amount of IaGV for ITS2 appears to be more than an order of magnitude greater for flowering plants than for the current sampling of *Haematococus* isolates. The average number of nucleotide variants for our data was 8 (R = 3–14) whereas the average number of nucleotide variants was 35 (R = 1–253) for the higher plant data [12]. PCR bias may be responsible for under-sampling of ITS2 haplotype variability in our analyses (see below). In addition, our data analysis pipeline excluded any variants/haplotypes that did not equal or exceed 1% of the total complement of filtered, merged and sampled reads. We selected this filter criterion since the majority of merged and sampled variants that fell below the 1% threshold did so at levels rarely exceeding 0.1% (5–15 reads per 10,000). This suggested that these minor variants should be regarded as background error. Had these sequences been included, the number of variants would have been higher and the two sets of data might have shown more congruence regarding IaGV. On the other hand, it remains possible that the differences in variant numbers reflect fundamental disparities in rRNA processing between members of distinct lineages within the Viridiplantae.

### Illumina sequencing vs clone data

While largely congruent with the results from Illumina deep sequencing, our clone data—which revealed six unique haplotypes for SAG 34-1h vs. three haplotypes from deep sequencing—suggest that the Illumina approach may be under-sampling the extent of ITS2 haplotype variability in at least some *Haematococcus*. The under-sampling possibility will need to be explored further by designing one or more alternative primer pairs for amplification of ITS2 from *Haematococcus* isolates. Additionally, further testing is warranted using the Illumina protocol by sequencing one or more of the flowering plant templates studied by Song *et al*. [12]. In this way, we can control for any differences in quality assurance and methodology.

### SAG 34-1h and SAG 34-1f

The observation that the SAG 34-1h and SAG 34-1f isolates bear ITS2 haplotypes that fall into different ITS2 clades is of special interest (Fig 2). One set of haplotype sequences is united with all isolates of the *H*. *pluvialis* alliance (Fig 2). The other sets of haplotype sequences—which includes the dominant haplotype—form the *H*. *rubens* clade [37] and the lineage “C” clade (Fig 2). The SAG 34-1h and the SAG 34-1f isolates bear the greatest number of detected nucleotide variants (14 and 10, respectively) among all isolates that were studied. Moreover, the average *p*-distance among haplotypes of the SAG 34-1h isolate is the highest and the average *p*-distance among haplotypes of the SAG 34-1f isolate is the next highest among all within-group comparisons (Table 2). These observations indicate that the phylogenetic disjunction between haplotypes is unlikely to be an artefact of short branches. The SAG 34-1f and SAG 34-1h isolates are also interesting in that they are characterized by a dominant haplotype with the highest frequency (0.98 and 0.95, respectively) among all comparisons (S1–S9 Figs).

Lastly, the clone data indicated that SAG 34-1h may possess more IaGV than was identified by the Illumina protocol (Table 6). Furthermore, at least one unique ITS2 haplotype from cloning was detected for all isolates except SAG 34-1c (Fig 2). We currently have no definitive explanation for the differences between clone data and the deep sequencing data. One likely explanation is PCR bias due to the use of different primer sets when targets were amplified for the two different approaches. Given the extent of primary sequence variability among ITS2 variants of SAG 34-1h, it is not unreasonable to expect that priming sites could exhibit variability.

Introgression is one possible explanation for the phylogenetically disjunct haplotypes of SAG 34-1h and SAG 34-1f. However, there is no evidence of a second parent lineage for either isolate even if we assume that one parent is from the *H*. *pluvialis* lineage. Although Triki *et al*. [52] reported gamete formation (see below), there is also little evidence that *H*. *pluvialis* or any of its allies has a functioning sexual cycle [35, 37]. Song *et al*. [12] argued that the rare instances of phylogenetically disjunct variants among the angiosperms represented evidence of molecular fossils from an earlier speciation event. Phylogenetic analysis shows that the SAG 34-1h haplotypes fall into separate clades that form a sister group with moderate bootstrap support (Fig 2). The phylogenetically disjunct haplotypes of SAG 34-1h appear to be an example of incomplete lineage sorting. On the other hand, the two haplotype lineages for SAG 34-1f are in different, non-monophyletic clades (Fig 2). If the SAG 34-1f haplotype lineages are the product of incomplete lineage sorting, then the corresponding speciation event would have to have occurred rather early in the diversification of *Haematococcus*. Nonetheless, a molecular fossil hypothesis makes the most sense given the phylogenetic results and the lack of evidence for hybridization in *Haematococcus*.

### Conclusions

From our observations of ITS2 variability in a small sample of green algae and the observations of ITS2 variability in angiosperms [12], we have concluded that additional assessments of intragenomic and intergenomic variation in other organisms are needed to fully address the potential consequences for phylogenetic analyses. Of equal or greater interest is the notion that intragenomic ITS2 variation could be useful as a tool for profiling organisms [53] or for identifying molecular relics of speciation [12] or introgression [54] in the evolutionary history of a lineage. Finally, our past work with *H*. *pluvialis* [35] has led us to ponder more fundamental issues associated with ITS2 and the rRNA cistron. If we assume that homogenization is reliant on processes predominantly associated with meiosis (e.g., unequal crossing over), then the case of *H*. *pluvialis* becomes quite intriguing. Unlike most other chlorophycean flagellates that exploit the hypnozygote as a means to survive environmental extremes, *H*. *pluvialis* relies on its vegetatively-produced akinete to survive desiccation or temperature extremes. Gamete formation in *H*. *pluvialis* has been observed, but syngamy and planozygote formation have been reported in only a few instances [52]. Neither zygote germination nor meiosis have been recorded. Pocock [55] concluded that “sexual reproduction is of very rare occurrence” in *H*. *pluvialis* and its putative allies. Given the apparent dearth of opportunities for recombination, one would predict that the rRNA cistron of *H*. *pluvialis*, absent the homogenizing influence of concerted evolution, is more likely to exhibit a relatively high level of intragenomic variability in contrast to organisms like *Chlamydomonas reinhardtii* whose dormant stages are produced via sexuality [56, 57].

Subsequent work will have several goals. One goal will be to optimize the modified Illumina protocol to minimize variability of variant frequency in replicate runs. In addition, we will test for undetected haplotypes by utilizing alternative priming during amplicon generation.

We also plan to compare deep sequencing results for green algae with deep sequencing of templates from higher plants where pyrosequencing identified several hundred nucleotide variants for some organisms. Lastly, we will begin testing biological hypotheses regarding the role of sex and meiosis in concerted evolution of the ribosomal RNA array. Does *Haematococcus* exhibit higher levels of IaGV than sexual organisms such as *Chlamydomonas*? Are sexual organisms less likely to bear ITS2 variants than asexual organisms? Or, do asexual organisms exploit other phenomena (e.g., homologous recombination during mitosis) to homogenize elements of the rRNA array?

## Supporting information

S1 Fig Intragenomic variants of ITS2 for isolate SAG 34-1b of *Haematococcus pluvialis*. Variants from three replicate sets of deep-sequencing are presented. Replicates 1 and 2 (R1 and R2) comprise results from sequencing of amplicons derived from the same template. Replicate 3 (R3) comprises results from sequencing of amplicons from a distinct template but extracted from the same isolate (SAG 34-1b). Variants, relative to the reference ITS2 sequence for SAG 34-1b (KC153465), are presented as both SNPs or DNPs (corresponding to specific sites in the reference and variant sequences) and as haplotypes (unique sets of SNPs that comprise whole ITS2 sequences). Relative frequencies (rounded to the nearest hundredth) for each SNP (or DNP) and for each haplotype are presented for each of the three replicates. Deletion sites are indicated as “DEL”.
(TIF)

S2 Fig Intragenomic variants of ITS2 for isolate SAG 34-1c of *Haematococcus rubicundus*. Variants from three replicate sets of deep-sequencing are presented. Replicates 1 and 2 (R1 and R2) comprise results from sequencing of amplicons derived from the same template. Replicate 3 (R3) comprises results from sequencing of amplicons from a distinct template but extracted from the same isolate (SAG 34-1c). Variants, relative to the reference ITS2 sequence for SAG 34-1c (KC153459), are presented as SNPs (corresponding to specific sites in the reference and variant sequences) and as haplotypes (unique sets of SNPs that comprise whole ITS2 sequences). Relative frequencies (rounded to the nearest hundredth) for each SNP and for each haplotype are presented for each of the three replicates. Deletion sites are indicated as “DEL”.
(TIF)

S3 Fig Intragenomic variants of ITS2 for isolate SAG 34-1f of *Haematococcus pluvialis*. Variants from two replicate sets of deep-sequencing are presented. Replicates 1 and 2 (R1 and R2) comprise results from sequencing of amplicons derived from the same template. Variants, relative to the reference ITS2 sequence for SAG 34-1f (KC153463), are presented as SNPs (corresponding to specific sites in the reference and variant sequences) and as haplotypes (unique sets of SNPs that comprise whole ITS2 sequences). Relative frequencies (rounded to the nearest hundredth) for each SNP and for each haplotype are presented for each of the replicates. Deletion sites are indicated as “DEL”.
(TIF)

S4 Fig Intragenomic variants of ITS2 for isolate SAG 34-1h of *Haematococcus rubens*. Variants from two replicate sets of deep-sequencing are presented. Replicates 1 and 2 (R1 and R2) comprise results from sequencing of amplicons derived from the same template. Nucleotide variants, relative to the reference ITS2 sequence for SAG 34-1h (KC153442), are presented as SNPs or DNPs (SNVs or MNVs corresponding to specific sites in the reference and variant sequences) and as haplotypes (unique sets of SNPs that comprise whole ITS2 sequences). Relative frequencies (rounded to the nearest hundredth) for each SNP and for each haplotype are presented for each of the replicates. Deletion sites are indicated as “DEL”. Phylogenetic analysis (Fig 2) shows that haplotypes 2 and 3 are more similar to isolates allied in the Pluvialis (A) lineage than they are to SAG 34-1h (KC153442).
(TIF)

S5 Fig Intragenomic variants of ITS2 for isolate SAG 34-1m of *Haematococcus rubicundus*. Variants from two replicate sets of deep-sequencing are presented. Replicates 1 and 2 (R1 and R2) comprise results from sequencing of amplicons derived from the same template. Nucleotide variants, relative to the reference ITS2 sequence for SAG 34-1m (KC153470), are presented as SNPs (corresponding to specific sites in the reference and variant sequences) and as haplotypes (unique sets of SNPs that comprise whole ITS2 sequences). Relative frequencies (rounded to the nearest hundredth) for each SNP and for each haplotype are presented for each of the three replicates. Deletion sites are indicated as “DEL”.
(TIF)

S6 Fig Intragenomic variants of ITS2 for isolate SAG 49.94 of *Haematococcus pluvialis*. Variants from two replicate sets of deep-sequencing are presented. Replicates 1 and 2 (R1 and R2) comprise results from sequencing of amplicons derived from the same template. Nucleotide variants, relative to the reference ITS2 sequence for SAG 49.94 (KC153462), are presented as SNPs (corresponding to specific sites in the reference and variant sequences) and as haplotypes (unique sets of SNPs that comprise whole ITS2 sequences). Relative frequencies (rounded to the nearest hundredth) for each SNP and for each haplotype are presented for each of the replicates.
(TIF)

S7 Fig Intragenomic variants of ITS2 for isolate SAG 44.96 of *Haematococcus pluvialis*. Variants from a single set of deep-sequencing are presented. Nucleotide variants, relative to the reference ITS2 sequence for SAG 44.96 (KC153460), are presented as SNPs (corresponding to specific sites in the reference and variant sequences) and as haplotypes (unique sets of SNPs that comprise whole ITS2 sequences). Relative frequencies (rounded to the nearest hundredth) for each SNP and for each haplotype are presented for each of the replicates. Haplotype 1 is identical to published ITS2 sequence KC153460 for SAG 44.96 except for one ambiguous site (highlighted in black) which was recorded as “N” at site 171 in the published sequence.
(TIF)

S8 Fig Intragenomic variants of ITS2 for isolate HP036 of *Haematococcus pluvialis*. Variants from a single set of deep-sequencing are presented. Nucleotide variants, relative to the reference ITS2 sequence for HP036 (KC153431), are presented as SNPs (corresponding to specific sites in the reference and variant sequences) and as haplotypes (unique sets of SNPs that comprise whole ITS2 sequences). Relative frequencies (rounded to the nearest hundredth) for each SNP and for each haplotype are presented for each of the replicates.
(TIF)

S9 Fig Intragenomic variants of ITS2 for extract C1066 of isolate HP111 from *Haematococcus pluvialis*. Variants from two replicate sets of deep-sequencing are presented. Replicates 1 and 2 (R1 and R2) comprise results from sequencing of amplicons derived from the same template. Nucleotide variants, relative to the reference ITS2 sequence for isolate HP111 (unpublished), are presented as SNPs (corresponding to specific sites in the reference and variant sequences) and as haplotypes (unique sets of SNPs that comprise whole ITS2 sequences). Relative frequencies (rounded to the nearest hundredth) for each SNP and for each haplotype are presented for each of the replicates.
(TIF)

S10 Fig Portion of electropherogram (Sequencher v4.9) from assembly of ITS2 sequence fragments (Sanger) for SAG 34-1b. Although deep sequencing-by-synthesis indicates that more than 40% of ITS2 variants are characterized by a “T” at site 61 (see S1 Fig), all of the Sanger fragments used to assemble the published sequence for SAG 34-1b were read as presenting a “G” (arrows) with little or no evidence of ambiguity at the site in question. Thus, the published ITS2 sequence (Sanger) recorded a “G” at site 61 for sequence submission (KC153465).
(TIF)

S11 Fig Portion of electropherogram (Sequencher v4.9) from assembly of ITS2 sequence fragments (Sanger) for SAG 34-1c. Although the three fragments manifest ambiguity corresponding to site 76 (arrow) of the published ITS2 sequence, the passage was recorded as “G” for sequence submission (KC153459) given the strength of signal for the “G” peak relative to the secondary “A” peak. The passage in question corresponds to variable site 76 from analysis of intragenomic variation (deep sequencing-by-synthesis; S2 Fig).
(TIF)

S12 Fig Portion of electropherogram (Sequencher v4.9) from assembly of ITS2 sequence fragments (Sanger) for SAG 34-1f. Although the three fragments manifest ambiguity corresponding to site 75 (arrow) of the published ITS2 sequence, the passage was recorded as “G” for sequence submission (KC153463) given the strength of signal for the “G” peak relative to the secondary “A” peak. The passage in question corresponds to variable site 75 from analysis of intragenomic variation (deep sequencing-by-synthesis; S3 Fig).
(TIF)

S13 Fig Portion of electropherograms (Sequencher v4.9) from assembly of ITS2 sequence fragments (Sanger) for SAG 34-1h. **a**. Although deep sequencing-by-synthesis indicates that 4–8% of ITS2 nucleotide variants are characterized by a “T” at site 18 (see S4 Fig), all Sanger fragments used to assemble the published sequence for SAG 34-1h were read as presenting a “C” (arrows) with little or no evidence of ambiguity at the site in question. Thus, the published ITS2 sequence (Sanger) recorded a “C” at site 61 for sequence submission (KC153442).
**b.** Although Sanger sequencing shows a possible ambiguity in one of the fragments (arrow; corresponding to site 22 of the dominant variant in S4 Fig), none of variants detected by deep sequencing-by-synthesis possessed a substitution at this site (S4 Fig). The published ITS2 sequence (Sanger) recorded a “T” at site 22 for sequence submission (KC153442) because the secondary peak (G) was weak or absent in the two fragments.
(TIF)

S14 Fig Portion of electropherograms (Sequencher v4.9) from assembly of ITS2 sequence fragments (Sanger) for SAG 34-1m. **a.** Although one of the two fragments manifests ambiguity corresponding to site 58 (arrow) of the published ITS2 sequence, the passage was recorded as “T” for sequence submission (KC153470) given the strength of signal for the “T” peak relative to the secondary “C” peak. The passage in question corresponds to variable site 58 from analysis of intragenomic variation (deep sequencing-by-synthesis; S5 Fig).
**b.** Both fragments manifest subtle ambiguity that begins at site 224 (arrows) of the published ITS2 sequence and continues for the remainder of the read. The passage was recorded as “ATGTACT” for sequence submission (KC153470) given the strength of signal for the primary peaks relative to the secondary peaks. The secondary peaks comprise the passage, “CATGTAC” (arrowheads), for the corresponding set of primary peaks. Thus, a careful analysis of the sequential ambiguity suggests that an indel is responsible for this pattern. Deep sequencing-by-synthesis confirms that one of the subordinate haplotypes (2) has an inserted “C” at what would be site 224 (deletion sites were arbitrarily mapped to site 220 in haplotypes 1, 3 and 4; see S5 Fig) and the remainder of the sequence is shifted for those haplotypes.
(TIF)

S15 Fig Portion of electropherogram (Sequencher v4.9) from assembly of ITS2 sequence fragments (Sanger) for SAG 49.94. Although the two fragments manifest ambiguity corresponding to site 104 (arrows) of the published ITS2 sequence, the passage was recorded as a “T” for sequence submission (KC153462) given the strength of signal for the “T” peak relative to the secondary “A” peak. A small tertiary “C” peak is also noted in the lower sequence fragment. The passage in question corresponds to variable site 104 from analysis of intragenomic variation (deep sequencing-by-synthesis; S6 Fig).
(TIF)

S16 Fig Portion of electropherograms (Sequencher v4.9) from assembly of ITS2 sequence fragments (Sanger) for SAG 44.96. **a.** Although the lower fragment clearly manifests ambiguity corresponding to sites 18 and 19 (arrows) of the published ITS2 sequence, the passage was recorded as “TA” for sequence submission (KC153460) given the relative strength of signal for the T and A peaks (arrows). The passage in question also corresponds to variable sites 18 and 19 from analysis of intragenomic variation (deep sequencing-by-synthesis; S7 Fig).
**b.** Ambiguous site (arrow) was recorded as “N” at site 171 for sequence submission (KC153460) and corresponds to variable site 171 from analysis of intragenomic variation (S7 Fig).
(TIF)

S17 Fig Portion of electropherograms (Sequencher v4.9) from assembly of ITS2 sequence fragments (Sanger) for HP036. **a.** Although the lower of the two fragments manifests ambiguity corresponding to sites 21 and 24 (arrows) of the published ITS2 sequence, the passage was recorded as a “C” and an “A” for sequence submission (KC153431) given the strength of signal for the”C” and “A” peaks relative to the secondary “T” and “C” peaks. The passage in question corresponds to variable sites 21 and 24 from analysis of intragenomic variation (deep sequencing-by-synthesis; S8 Fig).
**b.** Although the lower of the three fragments manifests ambiguity corresponding to site 191 (arrow) of the published ITS2 sequence, the passage was recorded as a “C” for sequence submission (KC153431) given the strength of signal for the “C” peak relative to the secondary “A” peak (however, deep-sequencing recorded a “T” as the subordinate polymorphism; S8 Fig). The passage in question corresponds to variable site 191 from analysis of intragenomic variation (deep sequencing-by-synthesis; S8 Fig).
(TIF)

S18 Fig Portion of electropherogram (Sequencher v4.9) from assembly of ITS2 sequence fragments (Sanger) for HP111. Although the three fragments manifest ambiguity (“C” or “T”) corresponding to site 69 (arrow) of the annotated ITS2 sequence (this sequence was unpublished prior to this investigation), the passage was recorded as a “T” for use in the reference sequence. The passage in question corresponds to variable site 69 from analysis of intragenomic variation (deep sequencing-by-synthesis; S9 Fig).
(TIF)
