# Supplementary figures and images for: Assessing intragenomic variation of the internal transcribed spacer two: Adapting the Illumina metagenomics protocol
Source: PLoS One. 2017 Jul 18;12(7):e0181491. doi: 10.1371/journal.pone.0181491 (PMC5515447; doi:10.1371/journal.pone.0181491)

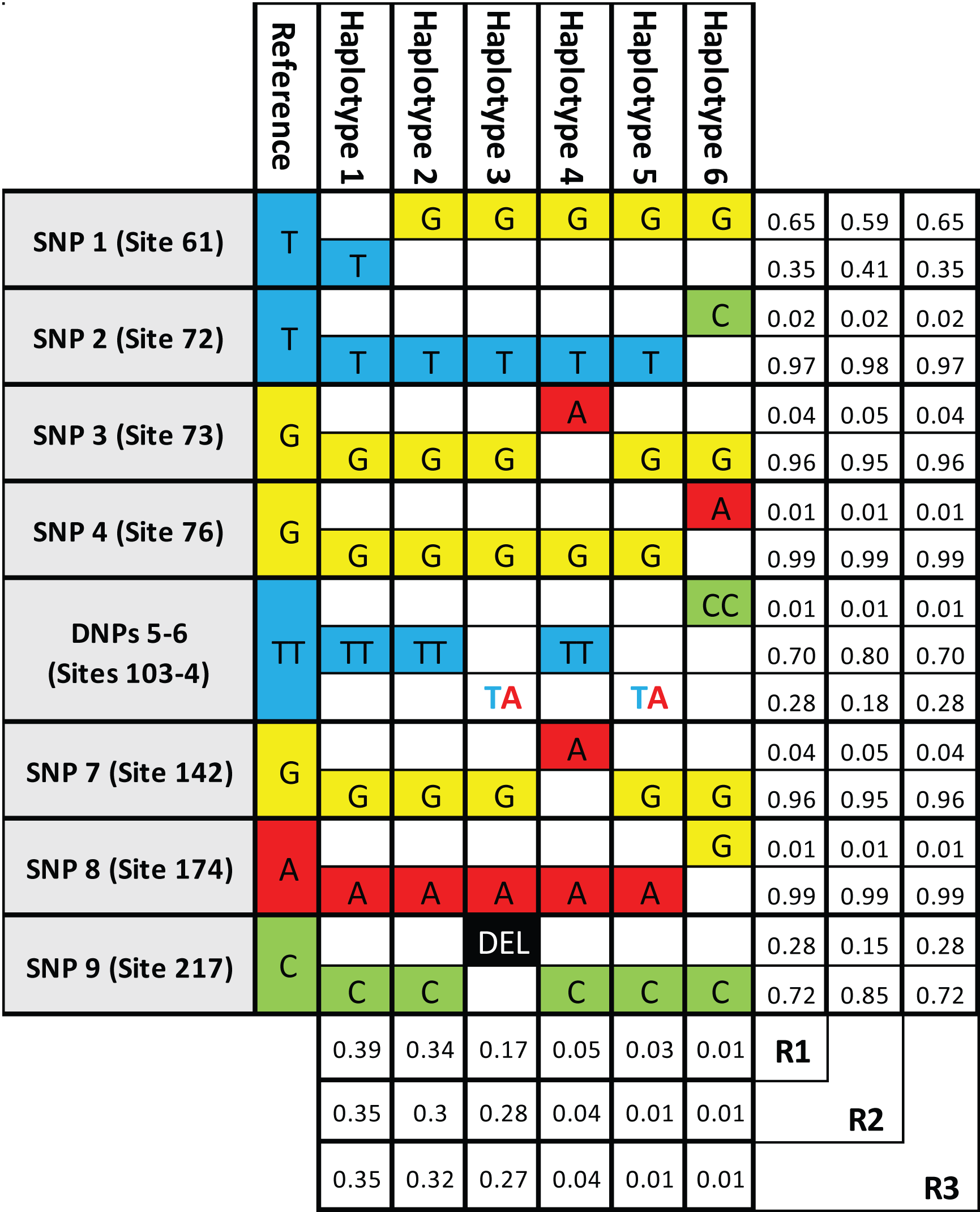

Supplement: S1 Fig — Variants from three replicate sets of deep-sequencing are presented. Replicates 1 and 2 (R1 and R2) comprise results from sequencing of amplicons derived from the same template. Replicate 3 (R3) comprises results from sequencing of amplicons from a distinct template but extracted from the same isolate (SAG 34-1b). Variants, relative to the reference ITS2 sequence for SAG 34-1b (KC153465), are presented as both SNPs or DNPs (corresponding to specific sites in the reference and variant sequences) and as haplotypes (unique sets of SNPs that comprise whole ITS2 sequences). Relative frequencies (rounded to the nearest hundredth) for each SNP (or DNP) and for each haplotype are presented for each of the three replicates. Deletion sites are indicated as “DEL”. (TIF) [file pone.0181491.s001.tif]

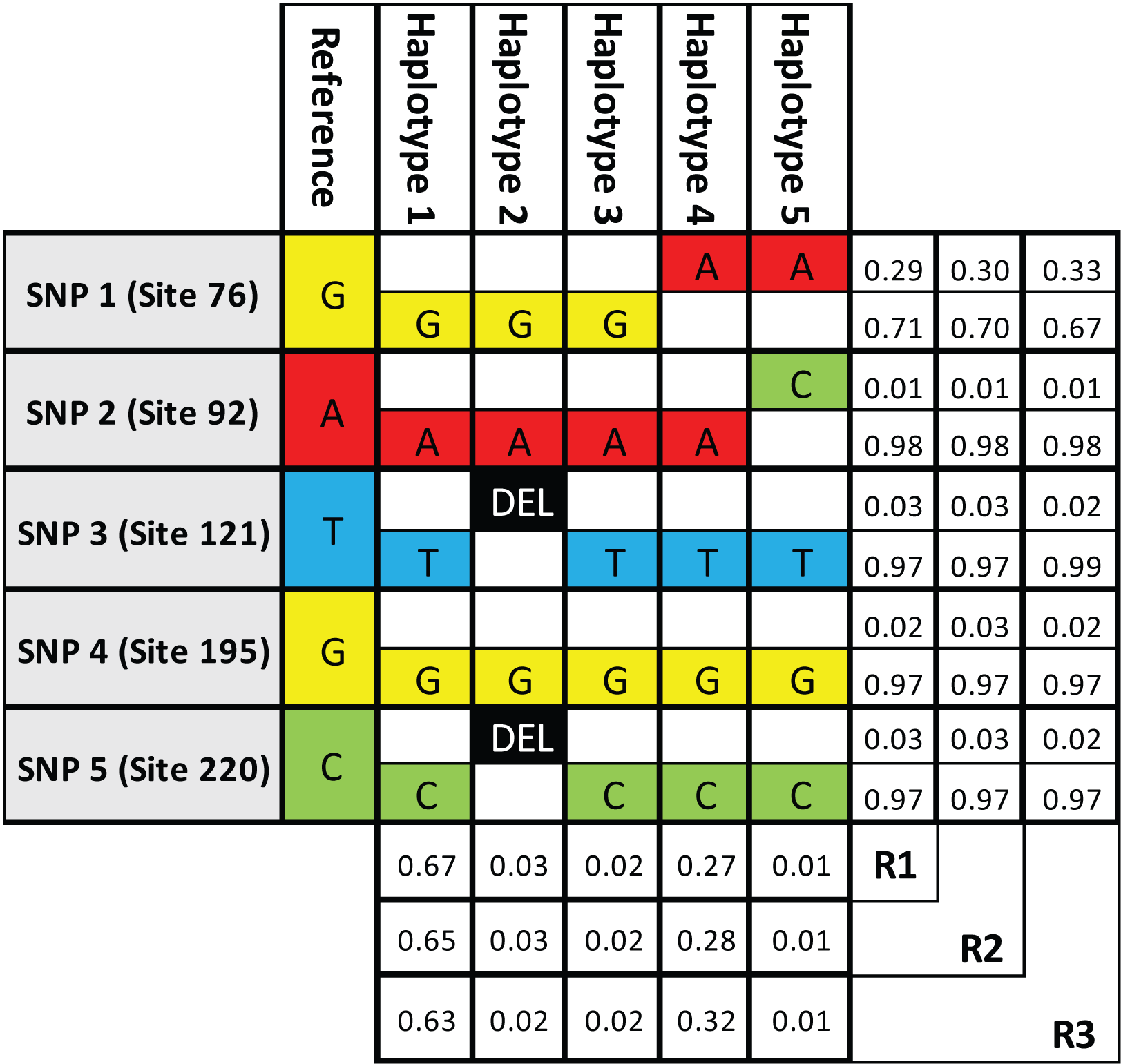

Supplement: S2 Fig — Variants from three replicate sets of deep-sequencing are presented. Replicates 1 and 2 (R1 and R2) comprise results from sequencing of amplicons derived from the same template. Replicate 3 (R3) comprises results from sequencing of amplicons from a distinct template but extracted from the same isolate (SAG 34-1c). Variants, relative to the reference ITS2 sequence for SAG 34-1c (KC153459), are presented as SNPs (corresponding to specific sites in the reference and variant sequences) and as haplotypes (unique sets of SNPs that comprise whole ITS2 sequences). Relative frequencies (rounded to the nearest hundredth) for each SNP and for each haplotype are presented for each of the three replicates. Deletion sites are indicated as “DEL”. (TIF) [file pone.0181491.s002.tif]

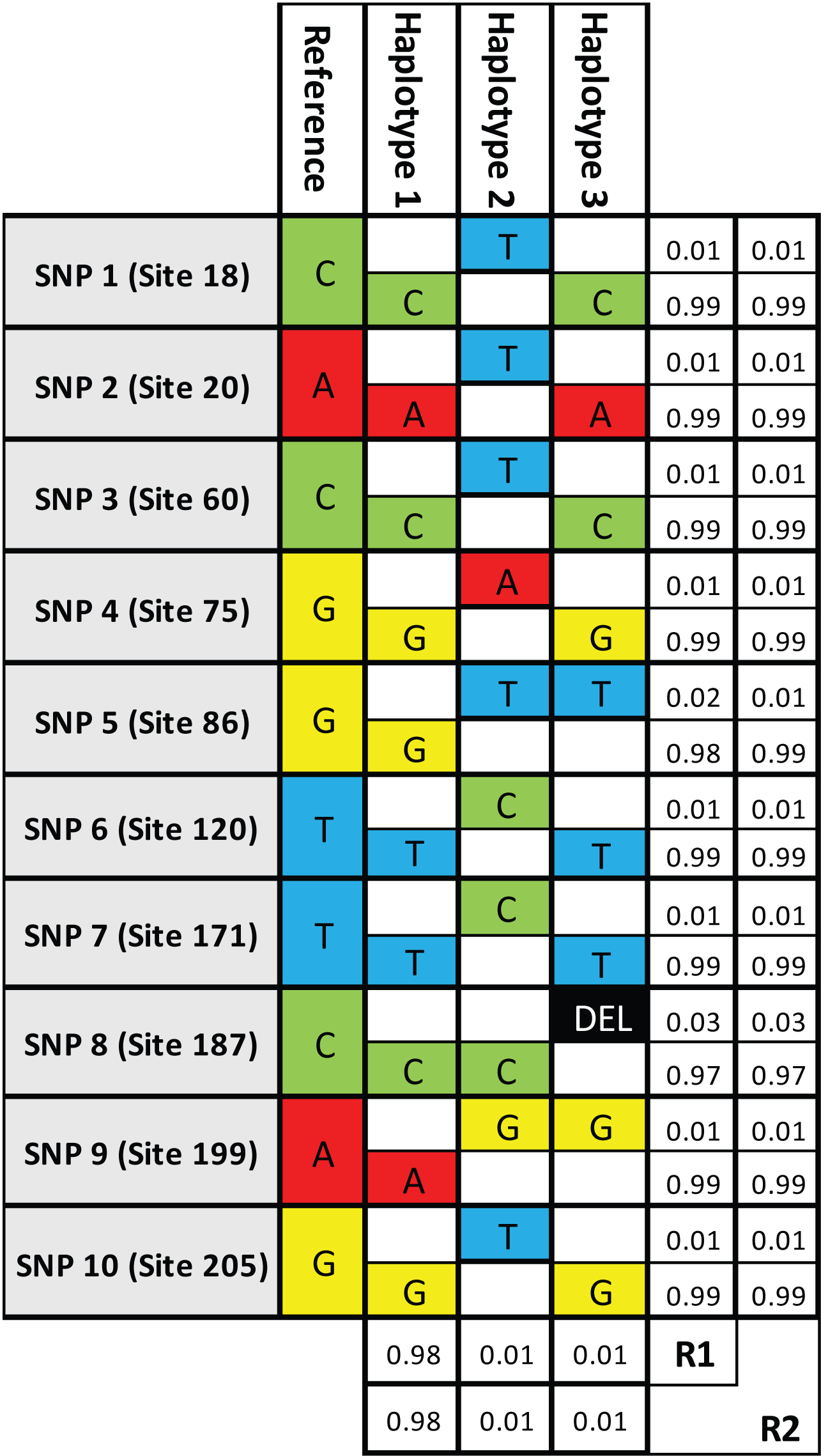

Supplement: S3 Fig — Variants from two replicate sets of deep-sequencing are presented. Replicates 1 and 2 (R1 and R2) comprise results from sequencing of amplicons derived from the same template. Variants, relative to the reference ITS2 sequence for SAG 34-1f (KC153463), are presented as SNPs (corresponding to specific sites in the reference and variant sequences) and as haplotypes (unique sets of SNPs that comprise whole ITS2 sequences). Relative frequencies (rounded to the nearest hundredth) for each SNP and for each haplotype are presented for each of the replicates. Deletion sites are indicated as “DEL”. (TIF) [file pone.0181491.s003.tif]

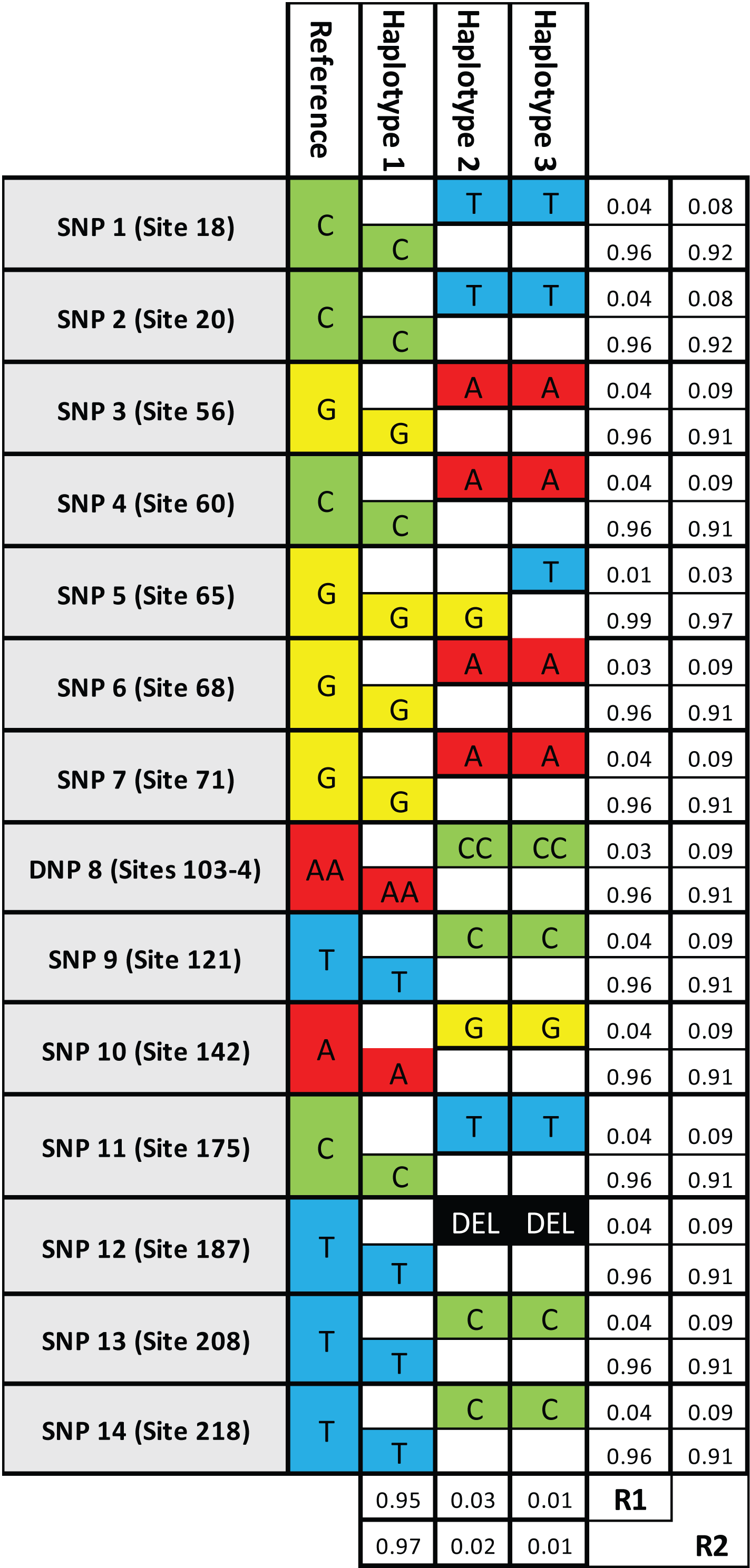

Supplement: S4 Fig — Variants from two replicate sets of deep-sequencing are presented. Replicates 1 and 2 (R1 and R2) comprise results from sequencing of amplicons derived from the same template. Nucleotide variants, relative to the reference ITS2 sequence for SAG 34-1h (KC153442), are presented as SNPs or DNPs (SNVs or MNVs corresponding to specific sites in the reference and variant sequences) and as haplotypes (unique sets of SNPs that comprise whole ITS2 sequences). Relative frequencies (rounded to the nearest hundredth) for each SNP and for each haplotype are presented for each of the replicates. Deletion sites are indicated as “DEL”. Phylogenetic analysis (Fig 2) shows that haplotypes 2 and 3 are more similar to isolates allied in the Pluvialis (A) lineage than they are to SAG 34-1h (KC153442). (TIF) [file pone.0181491.s004.tif]

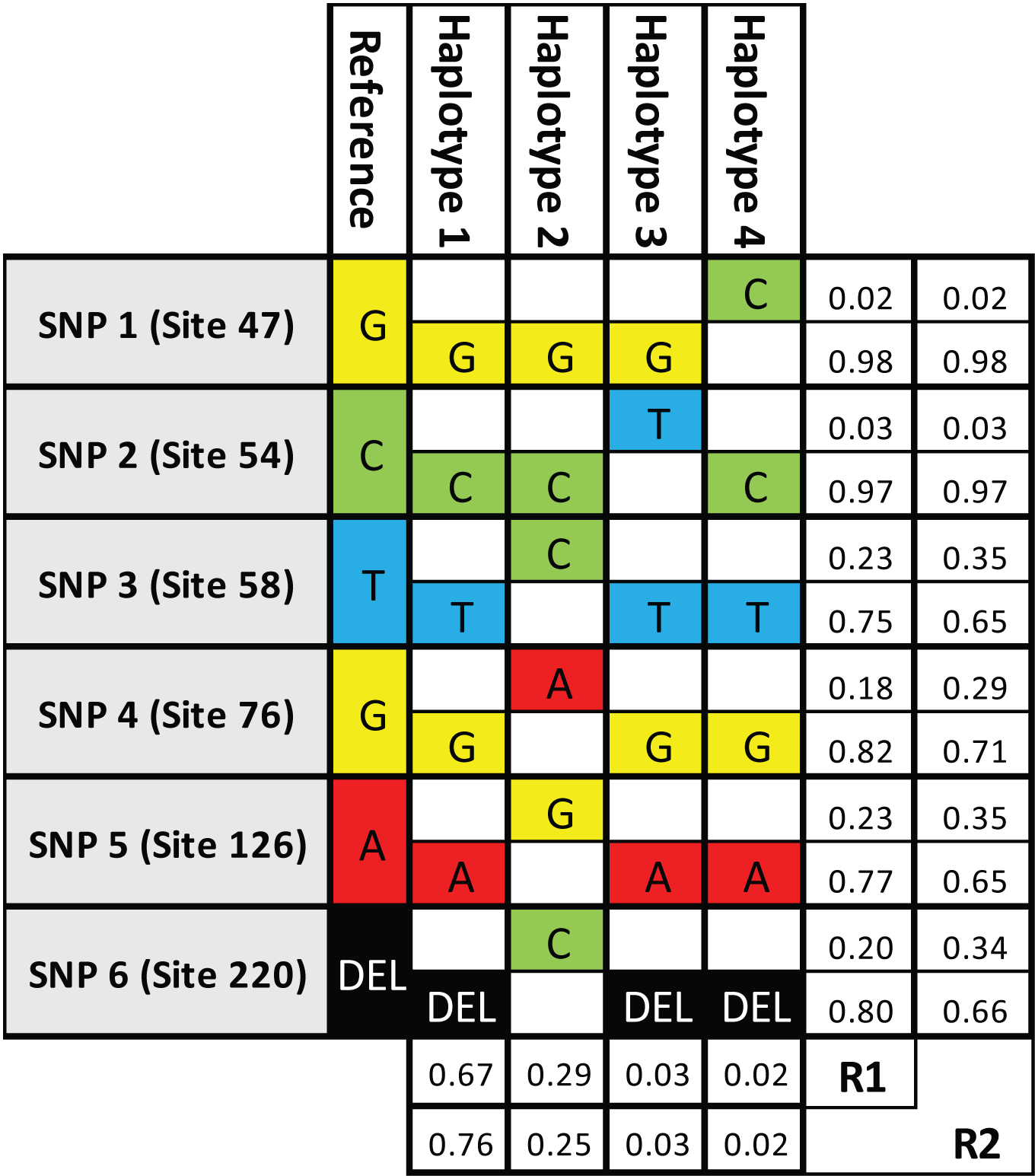

Supplement: S5 Fig — Variants from two replicate sets of deep-sequencing are presented. Replicates 1 and 2 (R1 and R2) comprise results from sequencing of amplicons derived from the same template. Nucleotide variants, relative to the reference ITS2 sequence for SAG 34-1m (KC153470), are presented as SNPs (corresponding to specific sites in the reference and variant sequences) and as haplotypes (unique sets of SNPs that comprise whole ITS2 sequences). Relative frequencies (rounded to the nearest hundredth) for each SNP and for each haplotype are presented for each of the three replicates. Deletion sites are indicated as “DEL”. (TIF) [file pone.0181491.s005.tif]

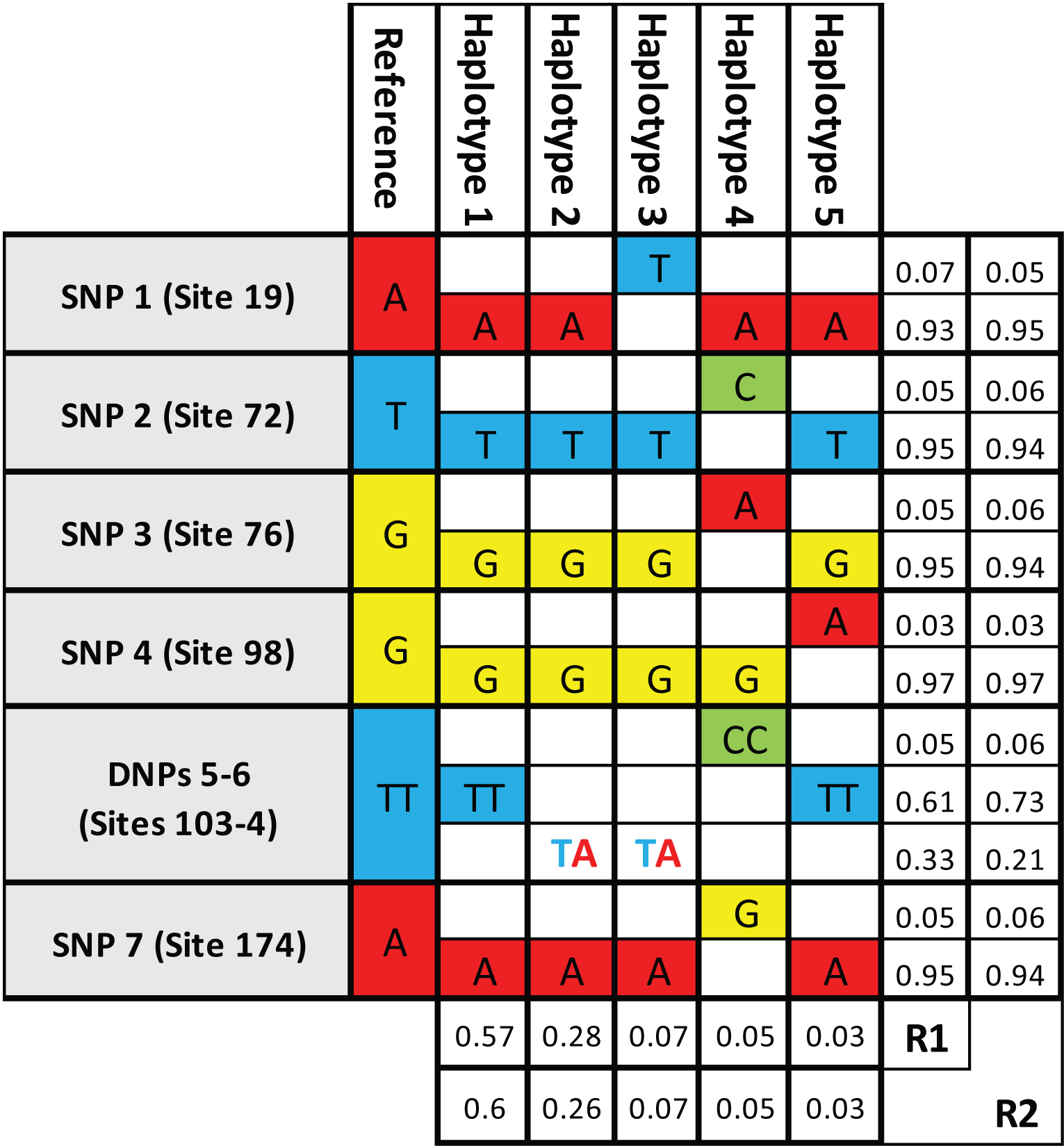

Supplement: S6 Fig — Variants from two replicate sets of deep-sequencing are presented. Replicates 1 and 2 (R1 and R2) comprise results from sequencing of amplicons derived from the same template. Nucleotide variants, relative to the reference ITS2 sequence for SAG 49.94 (KC153462), are presented as SNPs (corresponding to specific sites in the reference and variant sequences) and as haplotypes (unique sets of SNPs that comprise whole ITS2 sequences). Relative frequencies (rounded to the nearest hundredth) for each SNP and for each haplotype are presented for each of the replicates. (TIF) [file pone.0181491.s006.tif]

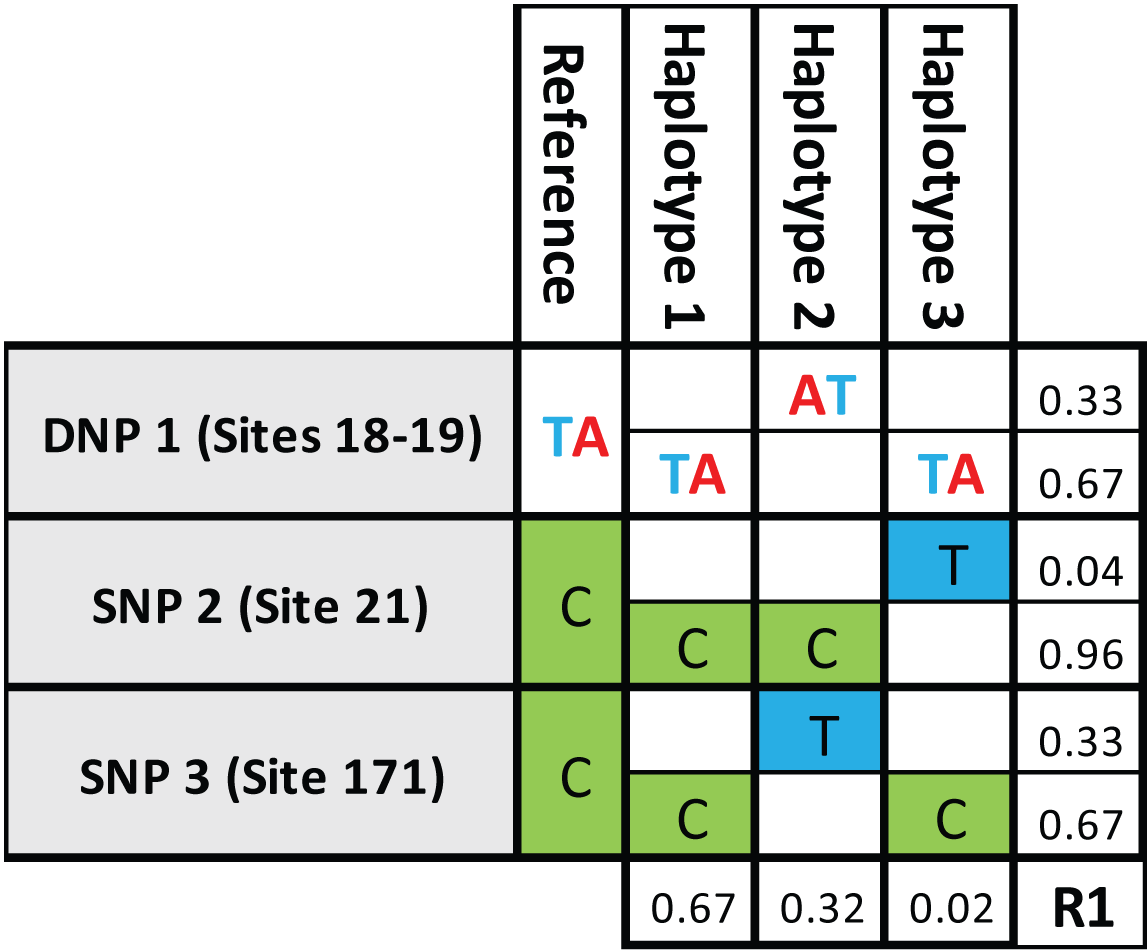

Supplement: S7 Fig — Variants from a single set of deep-sequencing are presented. Nucleotide variants, relative to the reference ITS2 sequence for SAG 44.96 (KC153460), are presented as SNPs (corresponding to specific sites in the reference and variant sequences) and as haplotypes (unique sets of SNPs that comprise whole ITS2 sequences). Relative frequencies (rounded to the nearest hundredth) for each SNP and for each haplotype are presented for each of the replicates. Haplotype 1 is identical to published ITS2 sequence KC153460 for SAG 44.96 except for one ambiguous site (highlighted in black) which was recorded as “N” at site 171 in the published sequence. (TIF) [file pone.0181491.s007.tif]

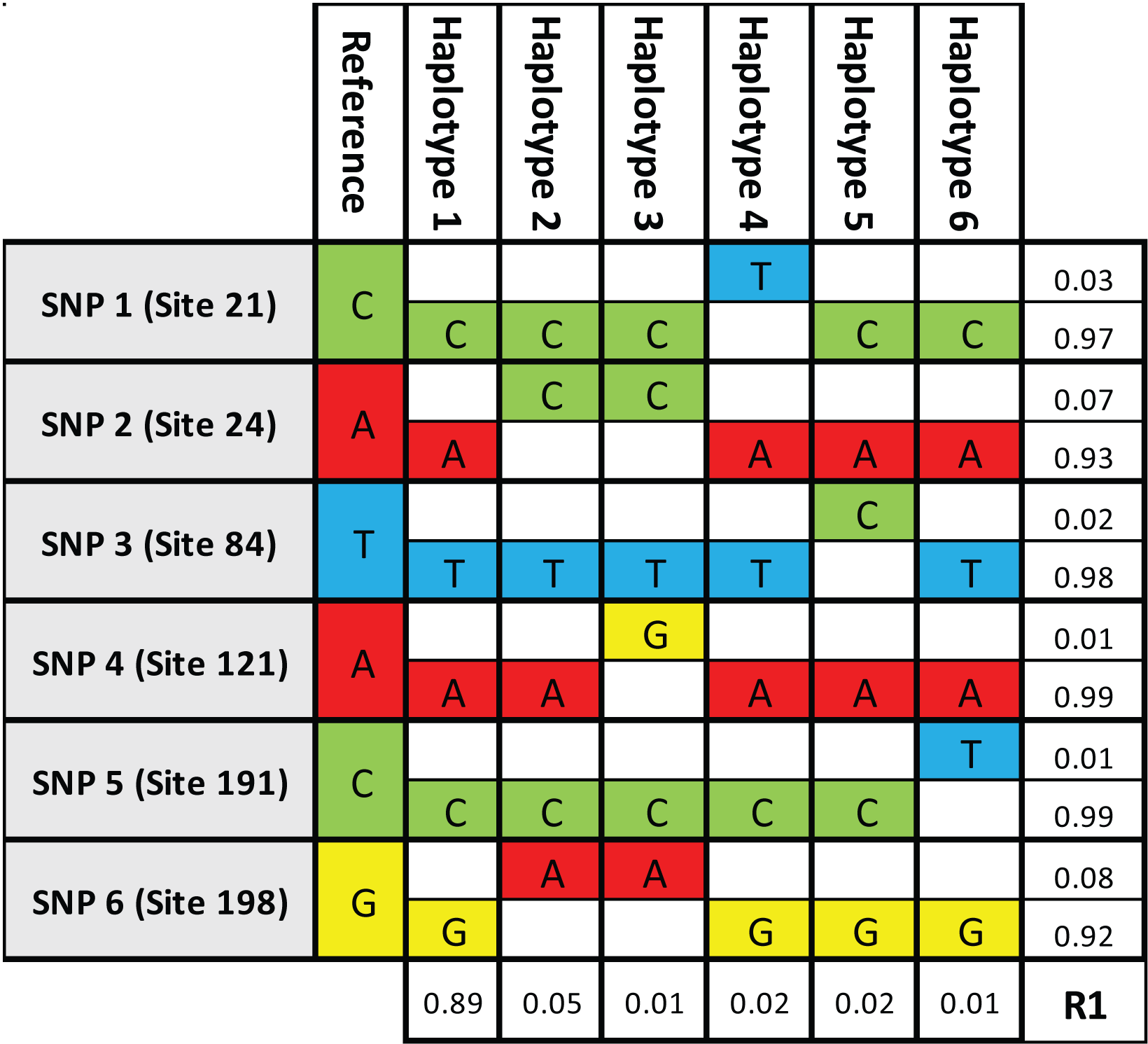

Supplement: S8 Fig — Variants from a single set of deep-sequencing are presented. Nucleotide variants, relative to the reference ITS2 sequence for HP036 (KC153431), are presented as SNPs (corresponding to specific sites in the reference and variant sequences) and as haplotypes (unique sets of SNPs that comprise whole ITS2 sequences). Relative frequencies (rounded to the nearest hundredth) for each SNP and for each haplotype are presented for each of the replicates. (TIF) [file pone.0181491.s008.tif]

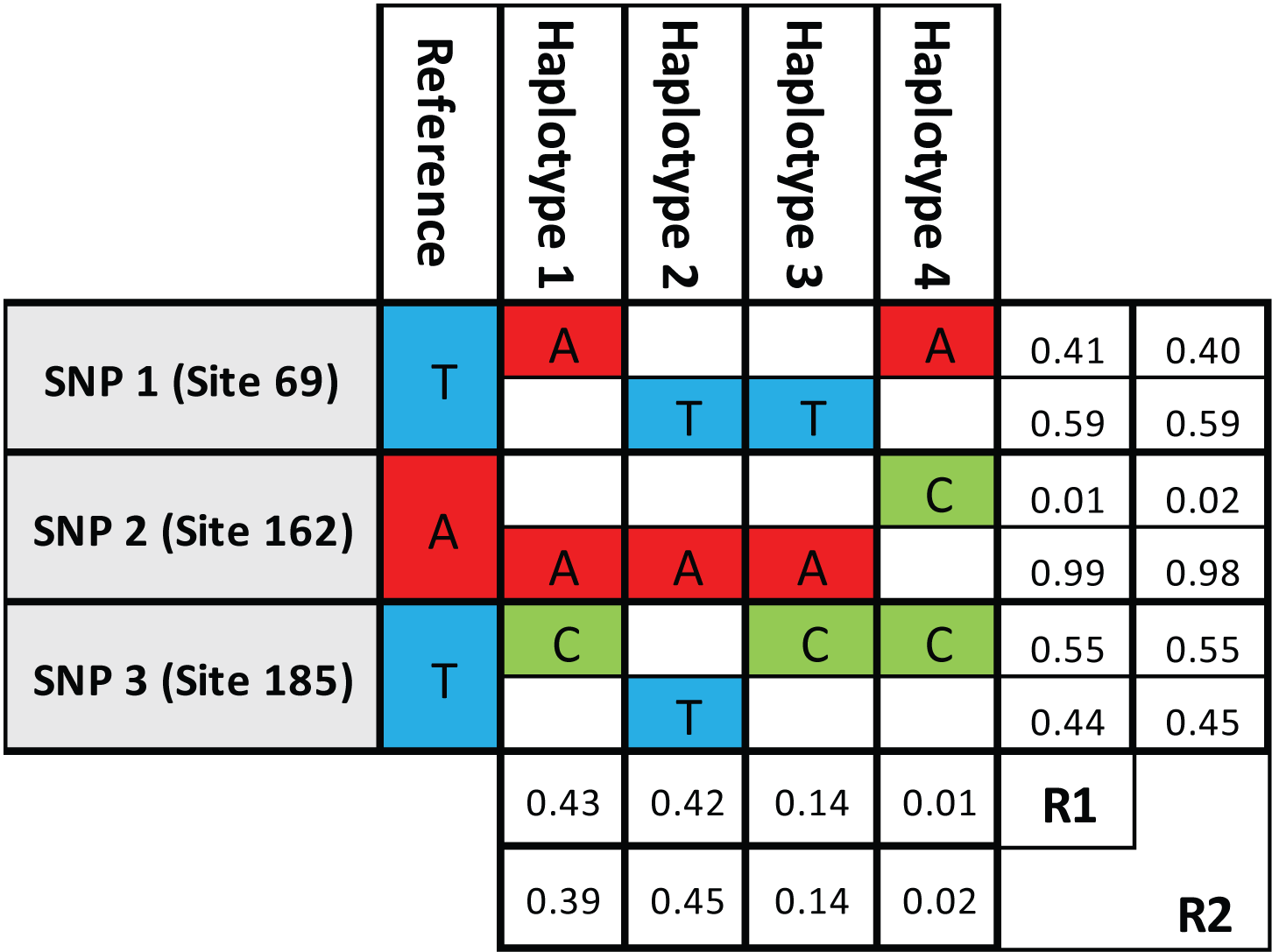

Supplement: S9 Fig — Variants from two replicate sets of deep-sequencing are presented. Replicates 1 and 2 (R1 and R2) comprise results from sequencing of amplicons derived from the same template. Nucleotide variants, relative to the reference ITS2 sequence for isolate HP111 (unpublished), are presented as SNPs (corresponding to specific sites in the reference and variant sequences) and as haplotypes (unique sets of SNPs that comprise whole ITS2 sequences). Relative frequencies (rounded to the nearest hundredth) for each SNP and for each haplotype are presented for each of the replicates. (TIF) [file pone.0181491.s009.tif]

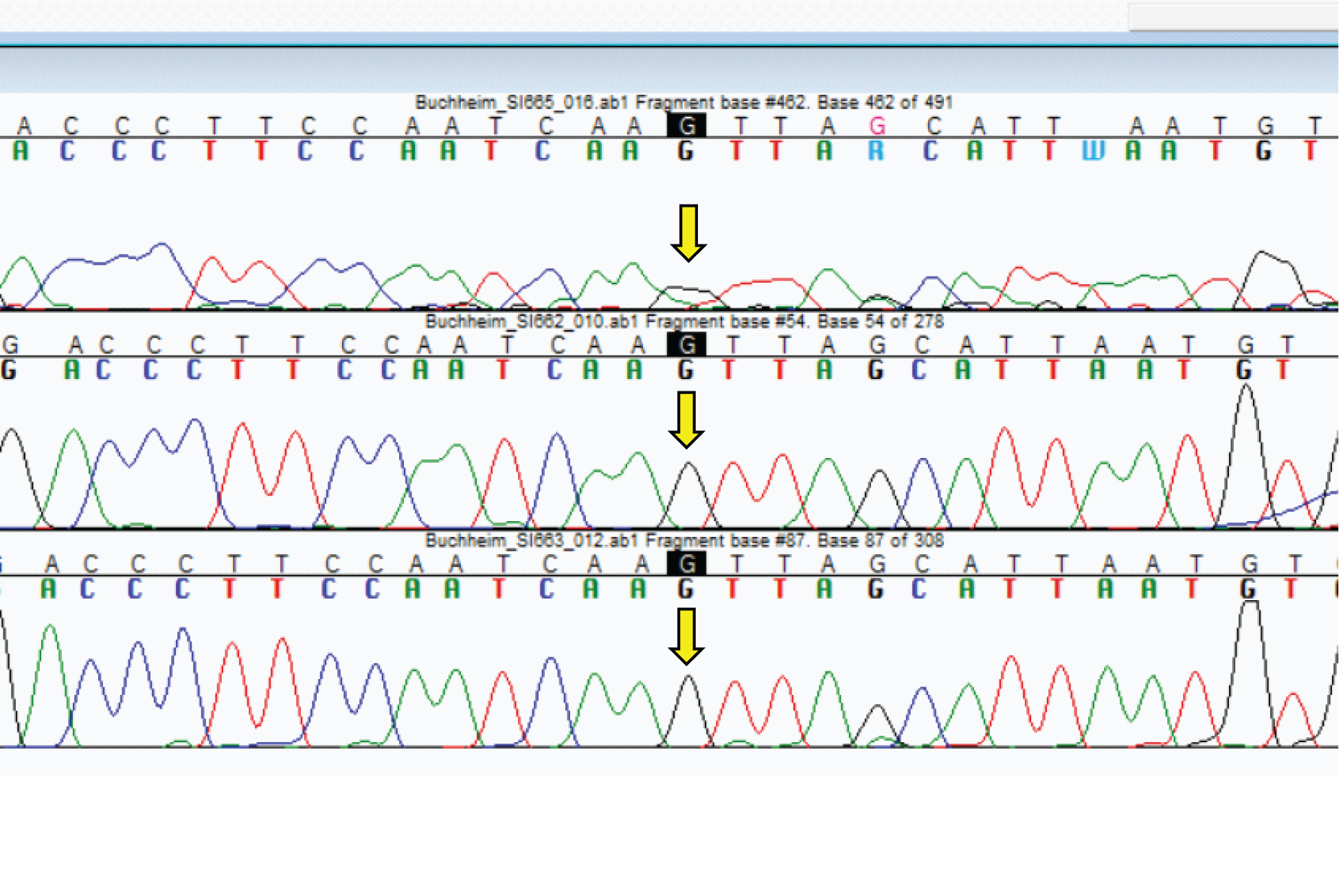

Supplement: S10 Fig — Although deep sequencing-by-synthesis indicates that more than 40% of ITS2 variants are characterized by a “T” at site 61 (see S1 Fig), all of the Sanger fragments used to assemble the published sequence for SAG 34-1b were read as presenting a “G” (arrows) with little or no evidence of ambiguity at the site in question. Thus, the published ITS2 sequence (Sanger) recorded a “G” at site 61 for sequence submission (KC153465). (TIF) [file pone.0181491.s010.tif]

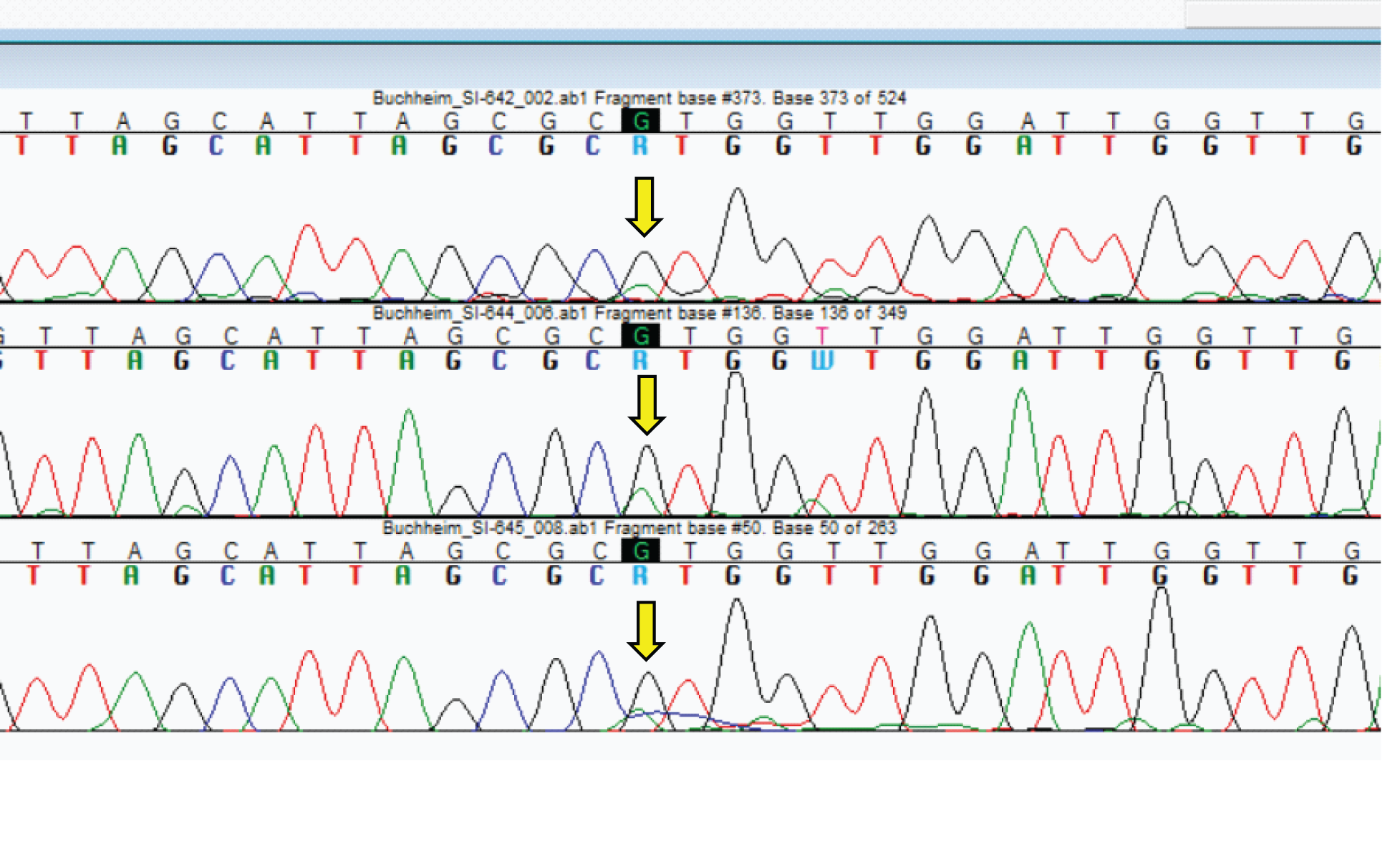

Supplement: S11 Fig — Although the three fragments manifest ambiguity corresponding to site 76 (arrow) of the published ITS2 sequence, the passage was recorded as “G” for sequence submission (KC153459) given the strength of signal for the “G” peak relative to the secondary “A” peak. The passage in question corresponds to variable site 76 from analysis of intragenomic variation (deep sequencing-by-synthesis; S2 Fig). (TIF) [file pone.0181491.s011.tif]

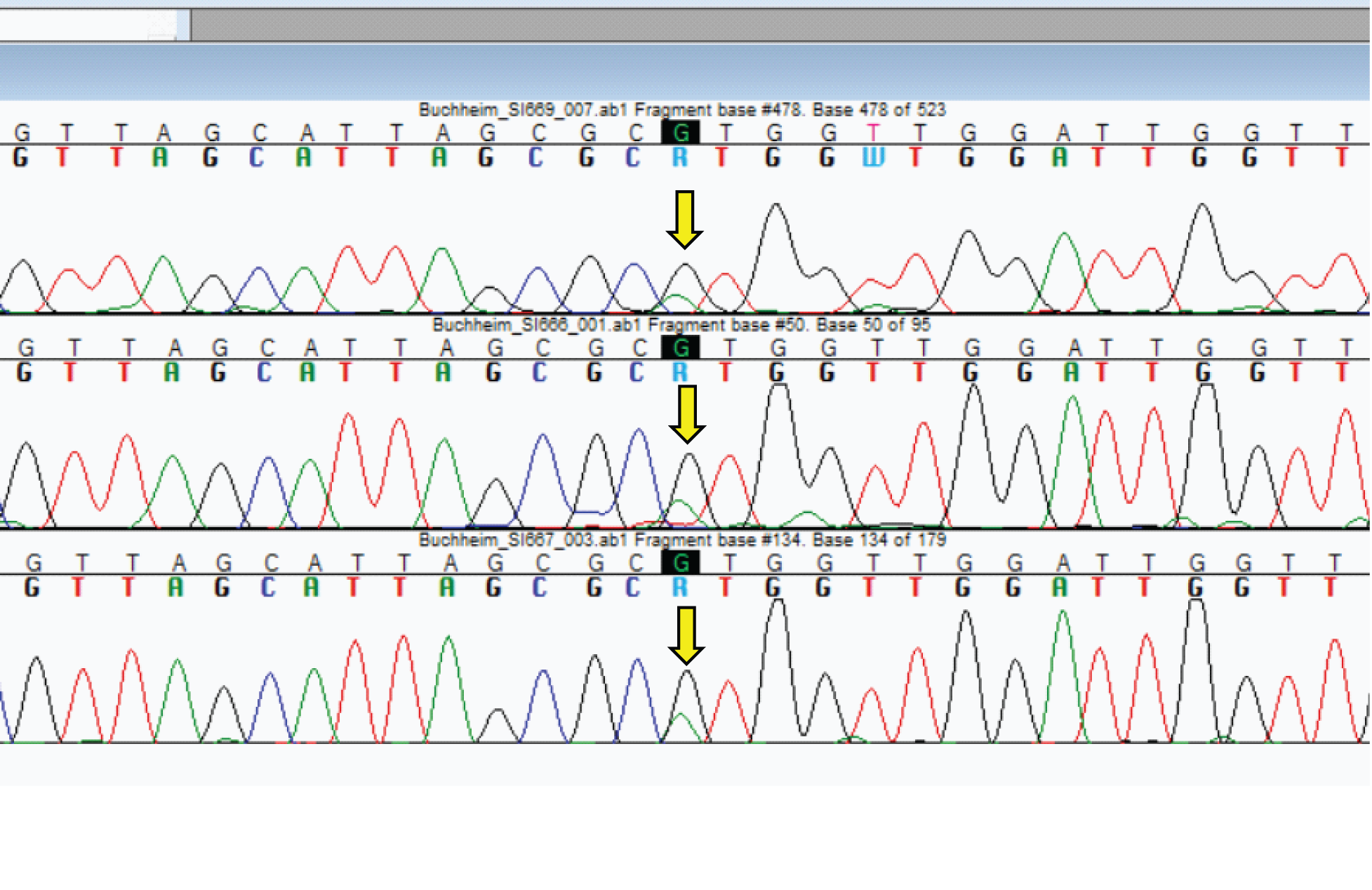

Supplement: S12 Fig — Although the three fragments manifest ambiguity corresponding to site 75 (arrow) of the published ITS2 sequence, the passage was recorded as “G” for sequence submission (KC153463) given the strength of signal for the “G” peak relative to the secondary “A” peak. The passage in question corresponds to variable site 75 from analysis of intragenomic variation (deep sequencing-by-synthesis; S3 Fig). (TIF) [file pone.0181491.s012.tif]

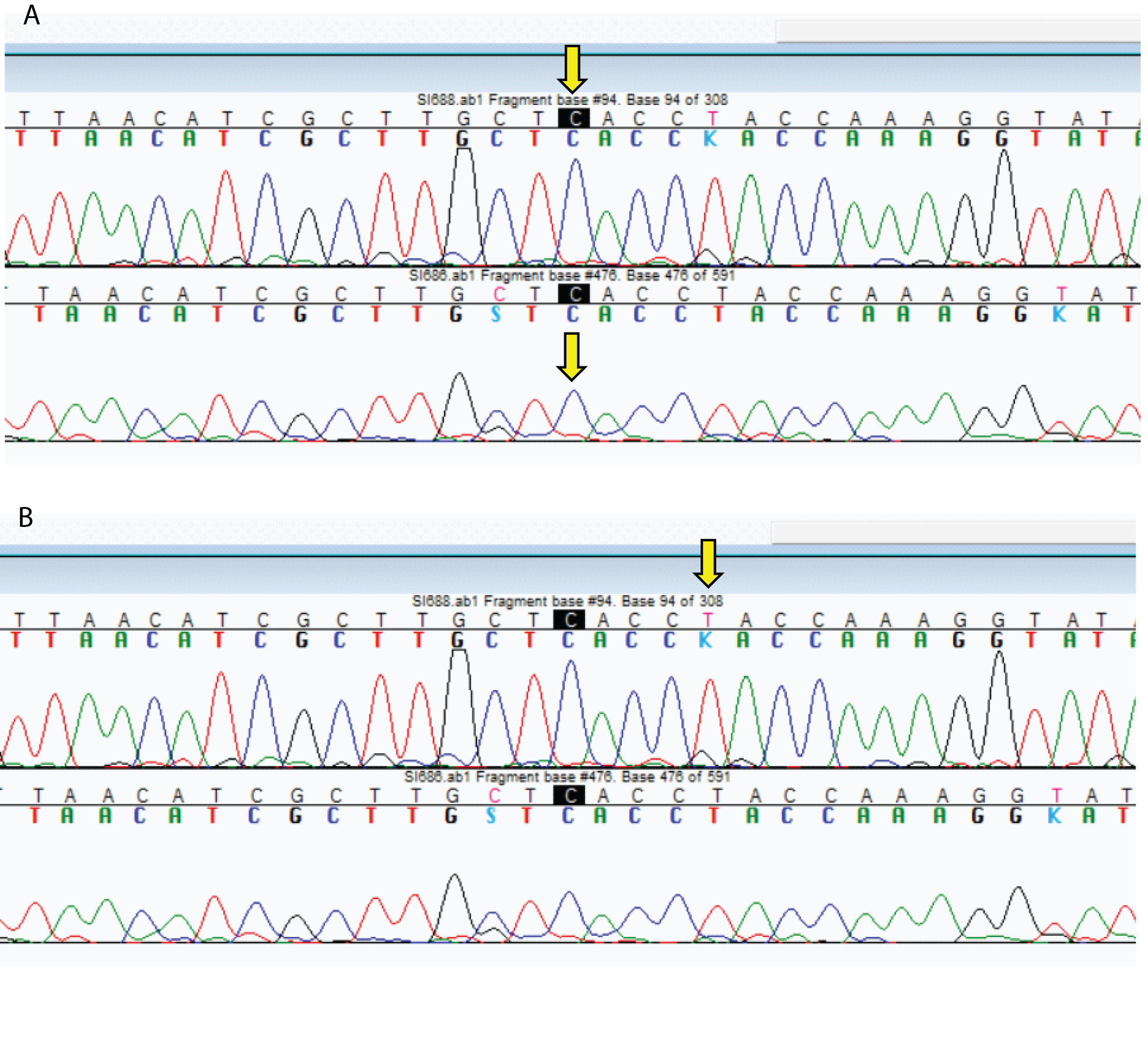

Supplement: S13 Fig — a. Although deep sequencing-by-synthesis indicates that 4–8% of ITS2 nucleotide variants are characterized by a “T” at site 18 (see S4 Fig), all Sanger fragments used to assemble the published sequence for SAG 34-1h were read as presenting a “C” (arrows) with little or no evidence of ambiguity at the site in question. Thus, the published ITS2 sequence (Sanger) recorded a “C” at site 61 for sequence submission (KC153442). b. Although Sanger sequencing shows a possible ambiguity in one of the fragments (arrow; corresponding to site 22 of the dominant variant in S4 Fig), none of variants detected by deep sequencing-by-synthesis possessed a substitution at this site (S4 Fig). The published ITS2 sequence (Sanger) recorded a “T” at site 22 for sequence submission (KC153442) because the secondary peak (G) was weak or absent in the two fragments. (TIF) [file pone.0181491.s013.tif]

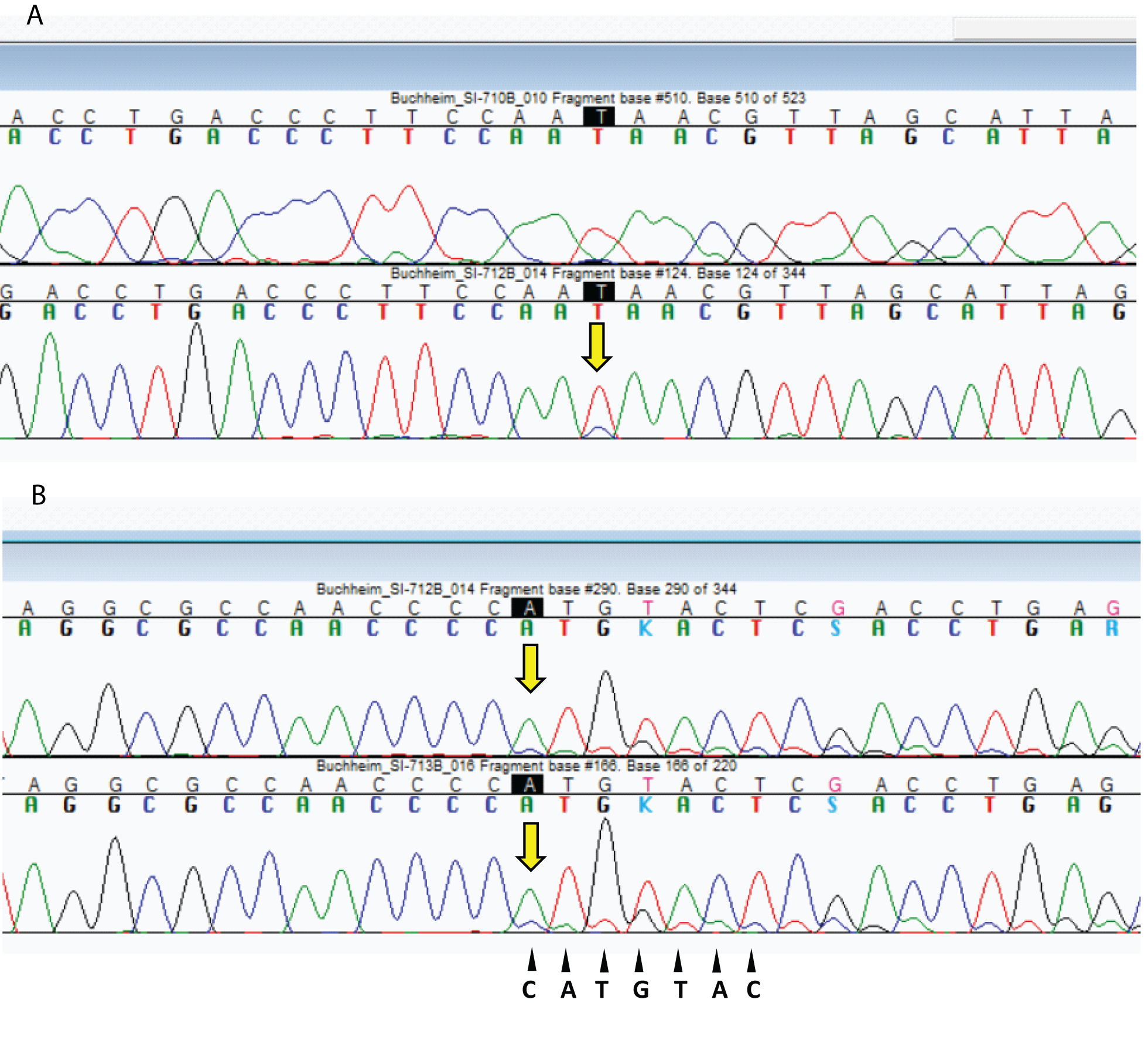

Supplement: S14 Fig — a. Although one of the two fragments manifests ambiguity corresponding to site 58 (arrow) of the published ITS2 sequence, the passage was recorded as “T” for sequence submission (KC153470) given the strength of signal for the “T” peak relative to the secondary “C” peak. The passage in question corresponds to variable site 58 from analysis of intragenomic variation (deep sequencing-by-synthesis; S5 Fig). b. Both fragments manifest subtle ambiguity that begins at site 224 (arrows) of the published ITS2 sequence and continues for the remainder of the read. The passage was recorded as “ATGTACT” for sequence submission (KC153470) given the strength of signal for the primary peaks relative to the secondary peaks. The secondary peaks comprise the passage, “CATGTAC” (arrowheads), for the corresponding set of primary peaks. Thus, a careful analysis of the sequential ambiguity suggests that an indel is responsible for this pattern. Deep sequencing-by-synthesis confirms that one of the subordinate haplotypes (2) has an inserted “C” at what would be site 224 (deletion sites were arbitrarily mapped to site 220 in haplotypes 1, 3 and 4; see S5 Fig) and the remainder of the sequence is shifted for those haplotypes. (TIF) [file pone.0181491.s014.tif]

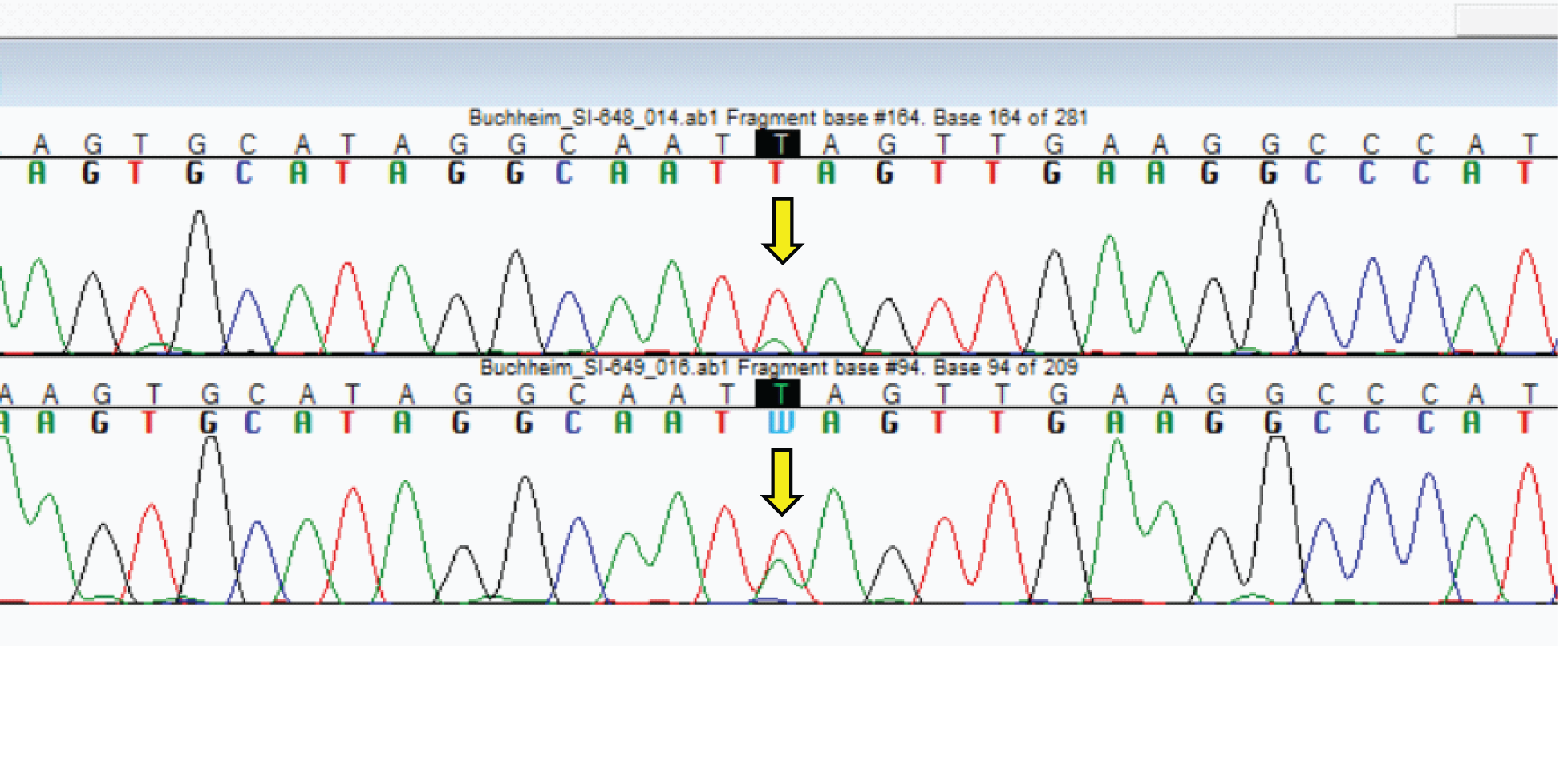

Supplement: S15 Fig — Although the two fragments manifest ambiguity corresponding to site 104 (arrows) of the published ITS2 sequence, the passage was recorded as a “T” for sequence submission (KC153462) given the strength of signal for the “T” peak relative to the secondary “A” peak. A small tertiary “C” peak is also noted in the lower sequence fragment. The passage in question corresponds to variable site 104 from analysis of intragenomic variation (deep sequencing-by-synthesis; S6 Fig). (TIF) [file pone.0181491.s015.tif]

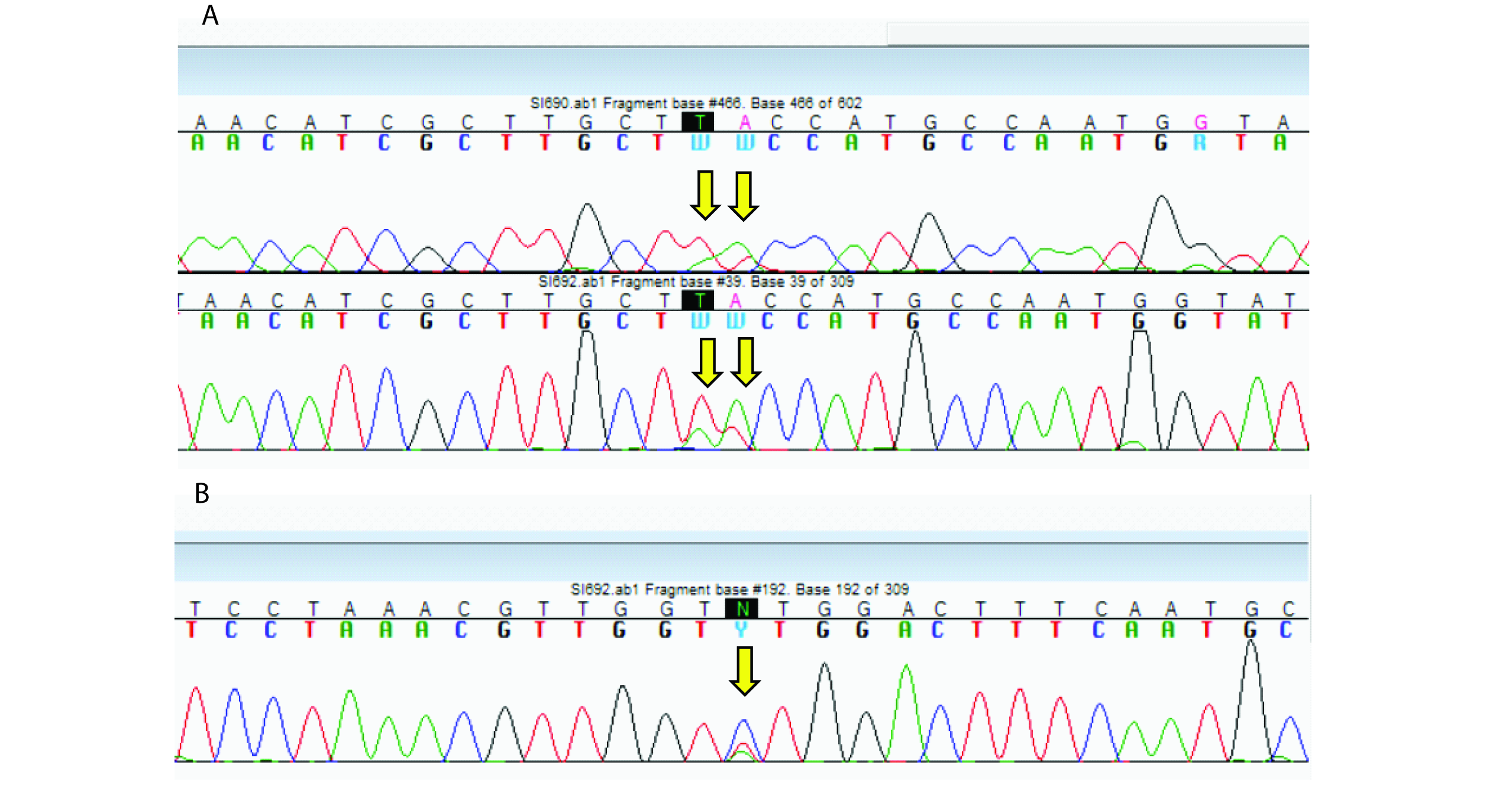

Supplement: S16 Fig — a. Although the lower fragment clearly manifests ambiguity corresponding to sites 18 and 19 (arrows) of the published ITS2 sequence, the passage was recorded as “TA” for sequence submission (KC153460) given the relative strength of signal for the T and A peaks (arrows). The passage in question also corresponds to variable sites 18 and 19 from analysis of intragenomic variation (deep sequencing-by-synthesis; S7 Fig). b. Ambiguous site (arrow) was recorded as “N” at site 171 for sequence submission (KC153460) and corresponds to variable site 171 from analysis of intragenomic variation (S7 Fig). (TIF) [file pone.0181491.s016.tif]

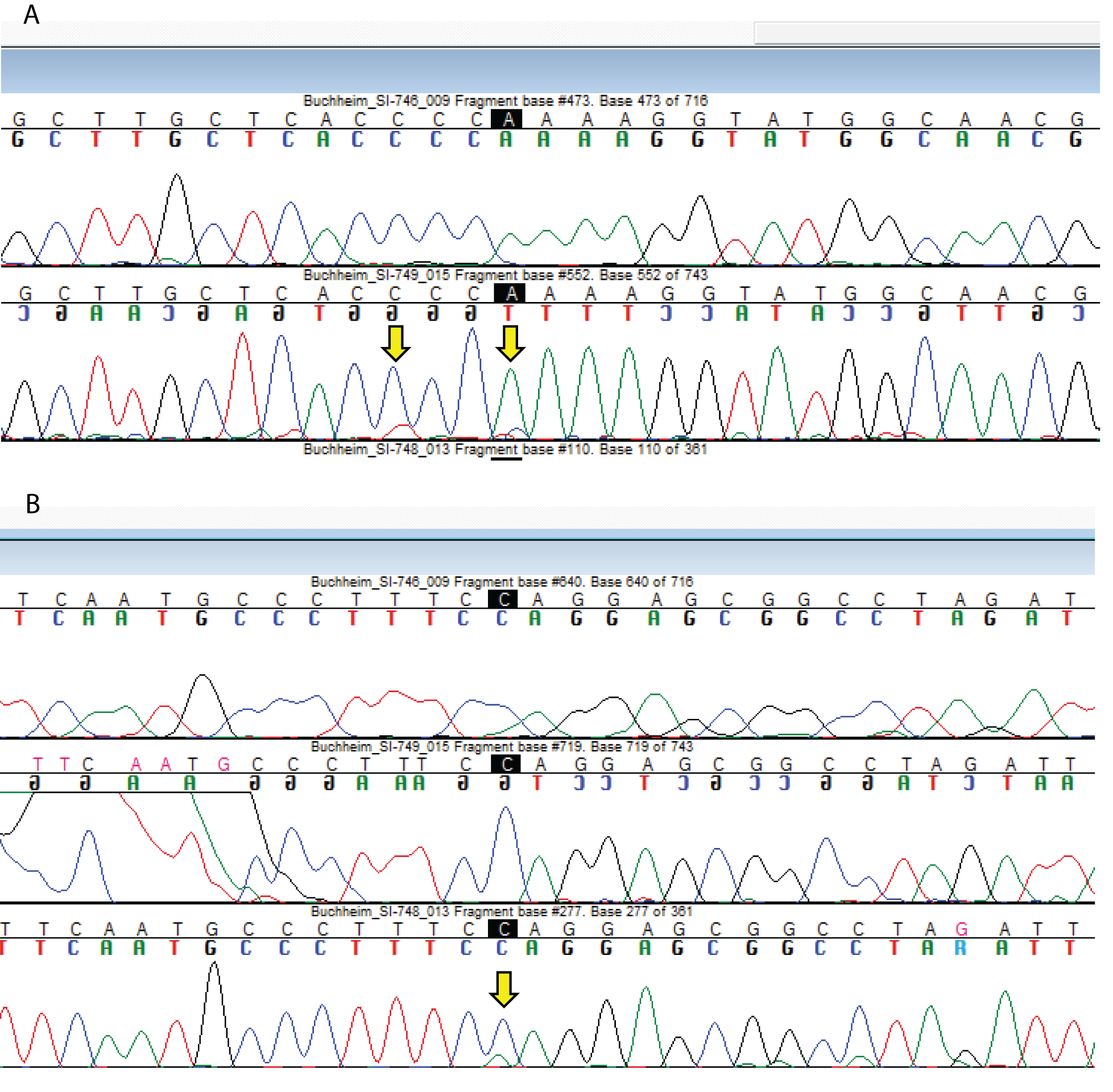

Supplement: S17 Fig — a. Although the lower of the two fragments manifests ambiguity corresponding to sites 21 and 24 (arrows) of the published ITS2 sequence, the passage was recorded as a “C” and an “A” for sequence submission (KC153431) given the strength of signal for the”C” and “A” peaks relative to the secondary “T” and “C” peaks. The passage in question corresponds to variable sites 21 and 24 from analysis of intragenomic variation (deep sequencing-by-synthesis; S8 Fig). b. Although the lower of the three fragments manifests ambiguity corresponding to site 191 (arrow) of the published ITS2 sequence, the passage was recorded as a “C” for sequence submission (KC153431) given the strength of signal for the “C” peak relative to the secondary “A” peak (however, deep-sequencing recorded a “T” as the subordinate polymorphism; S8 Fig). The passage in question corresponds to variable site 191 from analysis of intragenomic variation (deep sequencing-by-synthesis; S8 Fig). (TIF) [file pone.0181491.s017.tif]

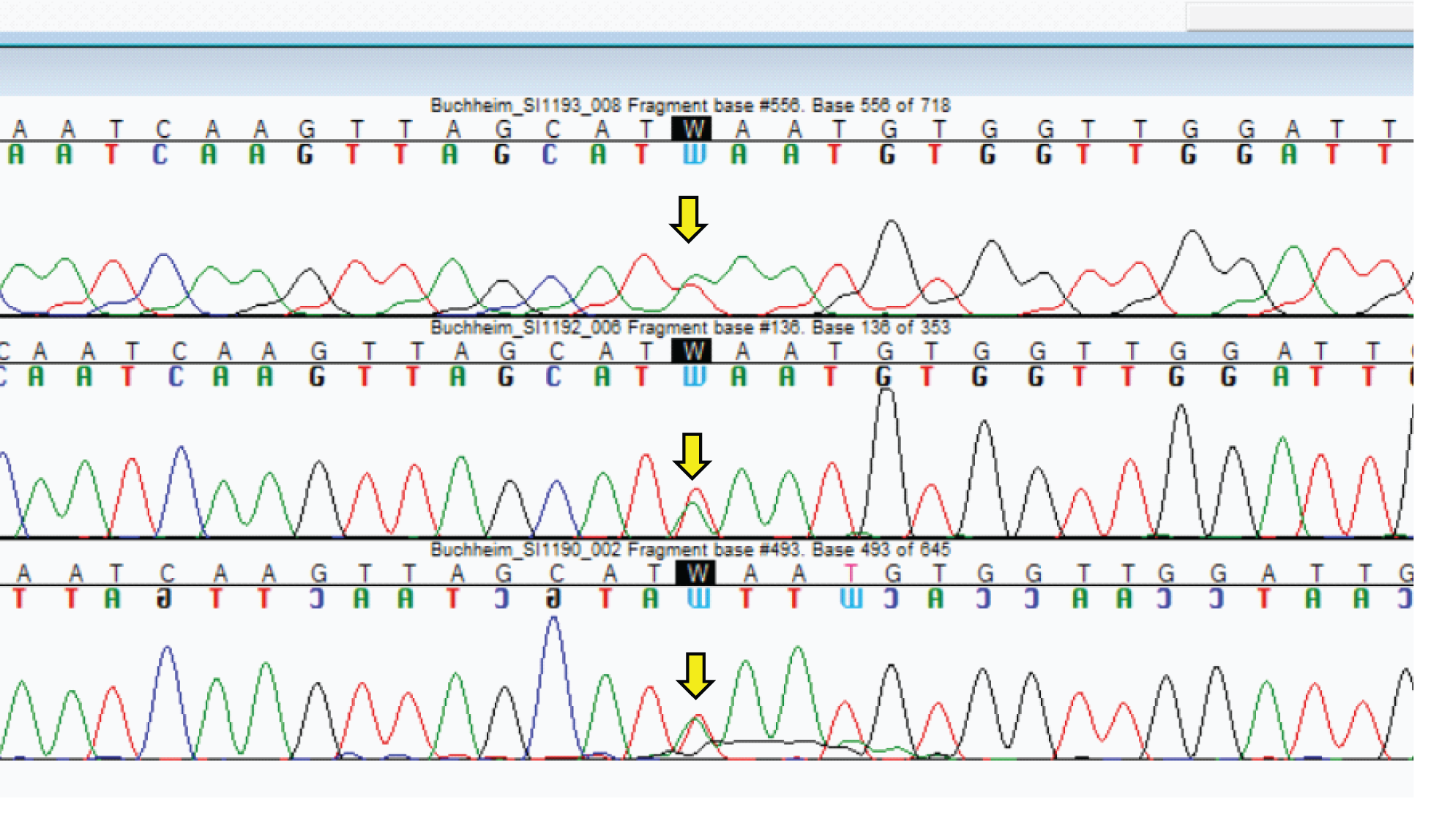

Supplement: S18 Fig — Although the three fragments manifest ambiguity (“C” or “T”) corresponding to site 69 (arrow) of the annotated ITS2 sequence (this sequence was unpublished prior to this investigation), the passage was recorded as a “T” for use in the reference sequence. The passage in question corresponds to variable site 69 from analysis of intragenomic variation (deep sequencing-by-synthesis; S9 Fig). (TIF) [file pone.0181491.s018.tif]
